# Tris{*N*-[bis(pyrrolidin-1-yl)phosphoryl]-2,2,2-trichloroacetamide}trichlorido­erbium(III)

**DOI:** 10.1107/S1600536810010408

**Published:** 2010-03-27

**Authors:** Kateryna O. Znovjyak, Vladimir A. Ovchynnikov, Svitlana V. Shishkina, Tetyana Yu. Sliva, Vladimir M. Amirkhanov

**Affiliations:** a Kyiv National Taras Shevchenko University, Department of Chemistry, Volodymyrska str. 64, 01601 Kyiv, Ukraine; b STC "Institute for Single Crystals", National Academy of Science of Ukraine, Lenina ave. 60, 61001, Khar’kov, Ukraine

## Abstract

The asymmetric unit of the title compound, [ErCl_3_(C_10_H_17_Cl_3_N_3_O_2_P)_3_], contains two independent mol­ecules. In each mol­ecule, the Er^III^ ion is six-coordinated in a slightly distorted octa­hedral ErO_3_Cl_3_ geometry with a *fac*-arrangement of the donor atoms. Intra­molecular N—H⋯Cl hydrogen bonds influence the mol­ecular conformations. Some of the pyrrolidine fragments in the *N*-[bis(pyrrolidin-1-yl)phosphoryl]-2,2,2-trichloroacetamide ligands are disordered over two conformations of equal occupancy. The unusually porous crystal packing exhibits voids of 162, 158 and 13 Å^3 ^and short inter­molecular Cl⋯O contacts of 2.876 (3) and 3.022 (4) Å.

## Related literature

For the synthesis and coordination properties of the *N*-[bis(pyrrolidin-1-yl)phosphoryl]-2,2,2-trichloroacetamide ligand, see: Znovjyak *et al.* (2009 ▶; 2010 ▶) and for a structural investigation, see: Gholivand *et al.* (2006 ▶). 
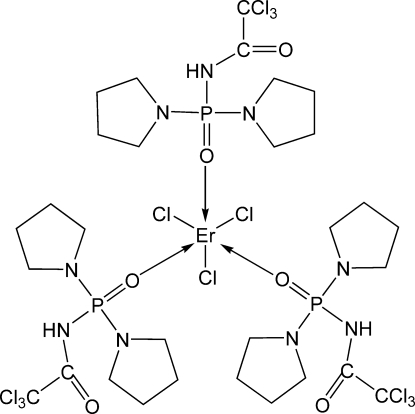


## Experimental

### 

#### Crystal data

[ErCl_3_(C_10_H_17_Cl_3_N_3_O_2_P)_3_]
*M* *_r_* = 1319.37
Triclinic, 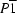

*a* = 15.285 (5) Å
*b* = 19.442 (5) Å
*c* = 20.283 (5) Å
α = 95.461 (5)°
β = 91.937 (5)°
γ = 106.405 (5)°
*V* = 5744 (3) Å^3^
*Z* = 4
Mo *K*α radiation
μ = 2.15 mm^−1^
*T* = 293 K
0.40 × 0.30 × 0.20 mm

#### Data collection

Oxford Diffraction Xcalibur3 diffractometer
Absorption correction: multi-scan (*CrysAlis RED*; Oxford Diffraction, 2006 ▶) *T*_min_ = 0.481, *T*_max_ = 0.674
76218 measured reflections
33091 independent reflections
16787 reflections with *I* > 2σ(*I*)
*R*_int_ = 0.036

#### Refinement

*R*[*F* ^2^ > 2σ(*F* ^2^)] = 0.040
*wR*(*F* ^2^) = 0.093
*S* = 0.89
33091 reflections
1135 parameters
67 restraints
H-atom parameters constrained
Δρ_max_ = 1.36 e Å^−3^
Δρ_min_ = −0.75 e Å^−3^

### 

Data collection: *CrysAlis CCD* (Oxford Diffraction, 2006 ▶); cell refinement: *CrysAlis RED* (Oxford Diffraction, 2006 ▶); data reduction: *CrysAlis RED*; program(s) used to solve structure: *SHELXS97* (Sheldrick, 2008 ▶); program(s) used to refine structure: *SHELXL97* (Sheldrick, 2008 ▶); molecular graphics: *ORTEP-3 for Windows* (Burnett & Johnson, 1996 ▶; Farrugia, 1997 ▶); software used to prepare material for publication: *WinGX* (Farrugia, 1999 ▶).

## Supplementary Material

Crystal structure: contains datablocks I, global. DOI: 10.1107/S1600536810010408/cv2699sup1.cif

Structure factors: contains datablocks I. DOI: 10.1107/S1600536810010408/cv2699Isup2.hkl

Additional supplementary materials:  crystallographic information; 3D view; checkCIF report

## Figures and Tables

**Table 1:** Hydrogen-bond geometry (Å, °)

*D*—H⋯*A*	*D*—H	H⋯*A*	*D*⋯*A*	*D*—H⋯*A*
N1*A*—H1*AA*⋯Cl11	0.86	2.48	3.247 (3)	149
N7*A*—H7*AC*⋯Cl12	0.86	2.43	3.234 (3)	157
N4*A*—H4*AC*⋯Cl13	0.86	2.50	3.248 (3)	147
N1*B*—H1*BA*⋯Cl21	0.86	2.44	3.214 (3)	150
N7*B*—H7*BC*⋯Cl22	0.86	2.52	3.293 (3)	149
N4*B*—H4*BC*⋯Cl23	0.86	2.52	3.295 (3)	150

**Fig. 1.**
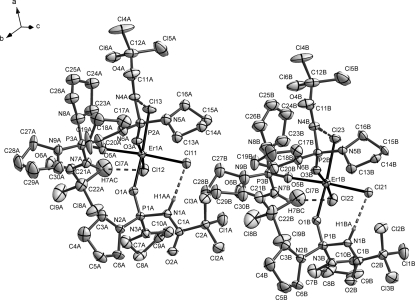
View of two crystallographically independent molecules in the unit cell of 1 with atom numbering scheme. Displacement ellipsoids are shown at 30% probability level. Dashed lines denote hydrogen bonds. Hydrogen atoms not involved in hydrogen bonding are omitted for clarity.

**Fig. 2.**
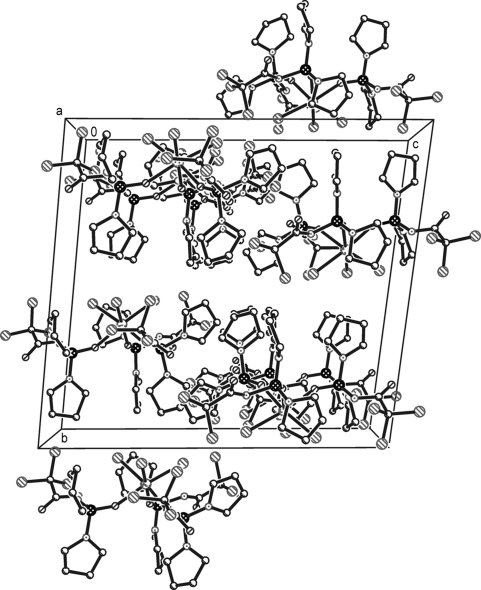
Projection of packing diagram of 1 along the a-direction.

## supplementary crystallographic information

### Comment

As a part of our study of coordination compounds based on carbacylamidophosphates with C(O)NHP(O) structural fragment we obtained the title compound [ErCl_3_*L*_3_] {*L* is *N*-[bis(pyrrolidin-1-yl)phosphoryl]-2,2,2-trichloroacetamide} (1), and determined its crystal structure. It has been shown previously that *L* is able to form complexes in the neutral and deprotonated form with lanthanides and uranium (Znovjyak *et al.*, 2009; Znovjyak *et al.*, 2010).

The asymmetric unit of the crystal structure of 1 contains two crystallographically independent molecules (Fig. 1 and 2). In each molecule erbium ion has six-coordinated environment formed by three O atoms of the P—O groups from three *L* molecules and by three chloride ions. The chloride ions additionally form intramolecular hydrogen bonding with the hydrogen atoms of the N—H groups of the *L* ligands (Table 2). The unusually porous crystal packing exhibits voids of 162, 158 and 13 Å^3^, respectively, and short intermolecular Cl···O contacts of 2.876 (3) and 3.022 (4) Å, respectively (Table 1). The coordination polyhedra of the Er^III^ ions are slightly distorted octahedra and represent *fac*-isomers. The phosphoryl group is situated in an *anti*-position with respect to the carbonyl group similarly to the crystal structure of the free ligand *L* described recently (Gholivand *et al.*, 2006). It was shown that *N*-[bis(pyrrolidin-1-yl)phosphoryl]-2,2,2-trichloroacetamide aggregates into the non-centrosymmetric dimers (*L*)_2_.

The Er—O and Er—Cl distances fall in the range 2.229 (2)-2.267 (2) Å and 2.594 (1)-2.615 (1) Å, respectively. Bond lengths in the fragment C(O)NHP(O) are slightly changed upon complexation. The P—N bond distances between phosphorus atoms and nitrogen atoms of the pyrrolidine substituents are shortened with respect to observed values in *L* (1.613 (4)-1.625 (4) Å) and fall in the range 1.569 (4)-1.613 (3) Å, that can be explained by increasing π-bonding in the (N_pyr_)_2_P(O) fragment due to complexation. The P—N(NH) bond lengths are shortened and P=O distances do not change upon ligand coordination and are equal to 1.685 (3)-1.693 (3) Å and 1.471 (3)-1.486 (3) Å, respectively. In the non-coordinated molecule (*L*)_2_, the C=O bond lengths are 1.192 (3) Å, 1.211 (3) Å, which in the case of 1 lie in the range of 1.184 (5)-1.218 (5) Å.

The environment of the phosphorus atoms in the coordinated *L* has a slightly distorted tetrahedral geometry where the highest deviation from the 109.28° is observed for the O—P—N angles. The environment of the amide nitrogen atoms is practically planar and the sum of the adjacent bond angles is equal to 359.9° indicating sp^2^-hybridization.

### Experimental

The synthesis of *L* was carried out according to the procedure described previously (Znovjyak *et al.*, 2009).

Hydrated chloride ErCl_3_6H_2_O (1 mmol) was solved upon heating in a mixture of isopropanol and methanol (2:1, 15 ml). The solution was dehydrated by HC(OC_2_H_5_)_3 _(6 mmol), then heated to the boiling point and cooled down. The resulting solution was added to the solution of *L* (3 mmol) in acetone (10 ml) and was left in a vacuum desiccator over CaCl_2_ at room temperature. After 1 day, the crystals of 1 were filtered off and washed with cold isopropanol and dried on the air (yield 85%). C_30_H_51_N_9_O_6_P_3_Cl_12_Er requires: Er, 12.68. Found: Er, 12.82%. IR (KBr, cm^-1^): 3150 ν(NH), 2980 ν(CH), 2895 ν(CH), 1740 ν(CO), 1445 ν(CN), 1220, 1155 ν(PO), 1100, 1030, 960, 890, 820, 680 ν(CCl).

### Refinement

All H atoms were placed at calculated positions and treated as riding on parent atoms [C—H=0.93 Å and *U*_iso_(H)=1.2*U*_eq_(C), N—H = 0.86 Å and *U*_iso_(H)=1.2*U*_eq_(N)]. Pyrrolidine fragments in both crystallographically independent molecules were treated as disordered over two conformations with the occupancies fixed to 0.5. We have not observed reflections with the theta angle < 2.92, that may be explained by technical limitations of the goniometer or by specific features of the crystal.

### Crystal data

**Table:**

[ErCl_3_(C_10_H_17_Cl_3_N_3_O_2_P)_3_]	*Z* = 4
*M**_r_* = 1319.37	*F*(000) = 2636
Triclinic, *P*1	*D*_x_ = 1.526 Mg m^−^^3^
*a* = 15.285 (5) Å	Mo *K*α radiation, λ = 0.71069 Å
*b* = 19.442 (5) Å	Cell parameters from 94170 reflections
*c* = 20.283 (5) Å	θ = 2.9–34.5°
α = 95.461 (5)°	µ = 2.15 mm^−^^1^
β = 91.937 (5)°	*T* = 293 K
γ = 106.405 (5)°	Block, rose
*V* = 5744 (3) Å^3^	0.40 × 0.30 × 0.20 mm

### Data collection

**Table:**

Oxford Diffraction Xcalibur3 diffractometer	33091 independent reflections
Radiation source: fine-focus sealed tube	16787 reflections with *I* > 2σ(*I*)
graphite	*R*_int_ = 0.036
Detector resolution: 16.1827 pixels mm^-1^	θ_max_ = 30.0°, θ_min_ = 2.9°
ω–scans	*h* = −21→21
Absorption correction: multi-scan (*CrysAlis RED*; Oxford Diffraction, 2006)	*k* = −27→27
*T*_min_ = 0.481, *T*_max_ = 0.674	*l* = −28→28
76218 measured reflections	

### Refinement

**Table:**

Refinement on *F*^2^	Primary atom site location: structure-invariant direct methods
Least-squares matrix: full	Secondary atom site location: difference Fourier map
*R*[*F*^2^ > 2σ(*F*^2^)] = 0.040	Hydrogen site location: inferred from neighbouring sites
*wR*(*F*^2^) = 0.093	H-atom parameters constrained
*S* = 0.89	*w* = 1/[σ^2^(*F*_o_^2^) + (0.0395*P*)^2^] where *P* = (*F*_o_^2^ + 2*F*_c_^2^)/3
33091 reflections	(Δ/σ)_max_ = 0.073
1135 parameters	Δρ_max_ = 1.36 e Å^−^^3^
67 restraints	Δρ_min_ = −0.75 e Å^−^^3^

### Special details

**Table:**

Geometry. All esds (except the esd in the dihedral angle between two l.s. planes) are estimated using the full covariance matrix. The cell esds are taken into account individually in the estimation of esds in distances, angles and torsion angles; correlations between esds in cell parameters are only used when they are defined by crystal symmetry. An approximate (isotropic) treatment of cell esds is used for estimating esds involving l.s. planes.
Refinement. Refinement of *F*^2^ against ALL reflections. The weighted *R*-factor wR and goodness of fit *S* are based on *F*^2^, conventional *R*-factors *R* are based on *F*, with *F* set to zero for negative *F*^2^. The threshold expression of *F*^2^ > σ(*F*^2^) is used only for calculating *R*-factors(gt) etc. and is not relevant to the choice of reflections for refinement. *R*-factors based on *F*^2^ are statistically about twice as large as those based on *F*, and *R*- factors based on ALL data will be even larger.

### Fractional atomic coordinates and isotropic or equivalent isotropic displacement parameters (Å^2^)

**Table:**

	*x*	*y*	*z*	*U*_iso_*/*U*_eq_	Occ. (<1)
Er1A	0.373766 (10)	−0.379006 (9)	0.179052 (8)	0.03818 (5)	
Cl11	0.28682 (7)	−0.45617 (6)	0.26773 (5)	0.0626 (3)	
Cl12	0.26251 (7)	−0.44632 (6)	0.07898 (5)	0.0606 (3)	
Cl13	0.49171 (7)	−0.45185 (6)	0.16092 (5)	0.0627 (3)	
Cl1A	0.06460 (9)	−0.47161 (7)	0.35223 (7)	0.0920 (4)	
Cl2A	0.03382 (10)	−0.36542 (10)	0.44965 (7)	0.1079 (5)	
Cl3A	0.21627 (8)	−0.37185 (8)	0.43059 (6)	0.0800 (4)	
Cl4A	0.88313 (10)	−0.33499 (14)	0.27668 (9)	0.1639 (10)	
Cl5A	0.72200 (16)	−0.45810 (10)	0.26900 (9)	0.1426 (8)	
Cl6A	0.75157 (9)	−0.36598 (9)	0.16658 (6)	0.0913 (4)	
Cl7A	0.26329 (14)	−0.44983 (9)	−0.10562 (9)	0.1296 (6)	
Cl8A	0.17538 (9)	−0.34697 (11)	−0.05369 (7)	0.1116 (6)	
Cl9A	0.24429 (12)	−0.33754 (12)	−0.18202 (7)	0.1308 (7)	
P1A	0.19763 (6)	−0.29365 (5)	0.22140 (5)	0.0409 (2)	
P2A	0.54955 (6)	−0.30029 (5)	0.31010 (5)	0.0391 (2)	
P3A	0.47175 (7)	−0.28467 (5)	0.04105 (5)	0.0452 (2)	
O1A	0.28407 (16)	−0.30591 (13)	0.19967 (12)	0.0479 (6)	
O2A	0.06750 (18)	−0.29163 (16)	0.33443 (13)	0.0589 (7)	
O3A	0.47266 (16)	−0.31249 (13)	0.25988 (12)	0.0452 (6)	
O4A	0.7500 (2)	−0.2863 (2)	0.34248 (16)	0.0825 (10)	
O5A	0.44500 (16)	−0.30285 (13)	0.10814 (12)	0.0494 (6)	
O6A	0.4079 (2)	−0.26954 (19)	−0.10219 (14)	0.0799 (10)	
N1A	0.18109 (19)	−0.33361 (16)	0.29236 (14)	0.0450 (7)	
H1AA	0.2169	−0.3589	0.3016	0.054*	
N2A	0.2082 (2)	−0.20873 (16)	0.22998 (17)	0.0534 (8)	
N3A	0.1039 (2)	−0.33192 (17)	0.17692 (15)	0.0500 (8)	
N4A	0.62702 (19)	−0.33151 (16)	0.26912 (14)	0.0462 (7)	
H4AC	0.6103	−0.3547	0.2304	0.055*	
N5A	0.5355 (2)	−0.3432 (2)	0.37441 (18)	0.0699 (6)	
N6A	0.5831 (2)	−0.21598 (17)	0.33648 (15)	0.0535 (8)	
N7A	0.3745 (2)	−0.32230 (17)	−0.00701 (15)	0.0504 (8)	
H7AC	0.3310	−0.3518	0.0108	0.060*	
N8A	0.5461 (2)	−0.3169 (2)	0.0066 (2)	0.0754 (7)	
N9A	0.5099 (2)	−0.19877 (18)	0.04296 (18)	0.0615 (9)	
C1A	0.1171 (2)	−0.3297 (2)	0.33591 (18)	0.0472 (9)	
C2A	0.1084 (3)	−0.3817 (2)	0.3901 (2)	0.0560 (11)	
C3A	0.2969 (3)	−0.1544 (2)	0.2361 (2)	0.0648 (12)	
H3AA	0.3336	−0.1633	0.1999	0.078*	0.50
H3AB	0.3295	−0.1553	0.2777	0.078*	0.50
H3CA	0.3209	−0.1505	0.1925	0.078*	0.50
H3CB	0.3406	−0.1661	0.2656	0.078*	0.50
C4A	0.2779 (14)	−0.0811 (6)	0.2336 (10)	0.092 (7)	0.50
H4AA	0.2762	−0.0686	0.1886	0.111*	0.50
H4AB	0.3232	−0.0429	0.2609	0.111*	0.50
C5A	0.1831 (12)	−0.0960 (4)	0.2622 (6)	0.084 (5)	0.50
H5AA	0.1893	−0.0868	0.3103	0.101*	0.50
H5AB	0.1497	−0.0651	0.2452	0.101*	0.50
C4C	0.2767 (10)	−0.0826 (5)	0.2565 (8)	0.061 (4)	0.50
H4CA	0.3180	−0.0431	0.2372	0.073*	0.50
H4CB	0.2812	−0.0717	0.3044	0.073*	0.50
C5C	0.1779 (10)	−0.0972 (4)	0.2279 (6)	0.078 (4)	0.50
H5CA	0.1481	−0.0647	0.2506	0.094*	0.50
H5CB	0.1761	−0.0921	0.1808	0.094*	0.50
C6A	0.1329 (3)	−0.1752 (2)	0.2407 (3)	0.0827 (15)	
H6AA	0.0948	−0.1966	0.2750	0.099*	0.50
H6AB	0.0951	−0.1805	0.2001	0.099*	0.50
H6CA	0.1113	−0.1775	0.2851	0.099*	0.50
H6CB	0.0819	−0.1976	0.2085	0.099*	0.50
C7A	0.0872 (4)	−0.3064 (3)	0.1119 (2)	0.0871 (16)	
H7AA	0.1359	−0.3073	0.0827	0.105*	0.50
H7AB	0.0807	−0.2580	0.1177	0.105*	0.50
H7CA	0.1396	−0.2791	0.0904	0.105*	0.50
H7CB	0.0498	−0.2752	0.1249	0.105*	0.50
C8A	−0.0031 (6)	−0.3621 (6)	0.0853 (7)	0.079 (5)	0.50
H8AA	−0.0549	−0.3549	0.1084	0.095*	0.50
H8AB	−0.0144	−0.3626	0.0379	0.095*	0.50
C9A	0.0209 (9)	−0.4301 (8)	0.1028 (4)	0.080 (5)	0.50
H9AA	−0.0325	−0.4720	0.0976	0.096*	0.50
H9AB	0.0681	−0.4398	0.0757	0.096*	0.50
C8C	0.0259 (13)	−0.3752 (9)	0.0715 (8)	0.151 (11)	0.50
H8CA	−0.0223	−0.3635	0.0466	0.181*	0.50
H8CB	0.0618	−0.3944	0.0401	0.181*	0.50
C9C	−0.0165 (8)	−0.4322 (11)	0.1176 (5)	0.094 (5)	0.50
H9CA	−0.0759	−0.4292	0.1306	0.112*	0.50
H9CB	−0.0217	−0.4807	0.0975	0.112*	0.50
C10A	0.0554 (3)	−0.4089 (2)	0.1759 (2)	0.0678 (12)	
H10A	0.0050	−0.4163	0.2048	0.081*	0.50
H10B	0.0962	−0.4360	0.1890	0.081*	0.50
H10C	0.0214	−0.4098	0.2154	0.081*	0.50
H10D	0.0931	−0.4412	0.1787	0.081*	0.50
C11A	0.7140 (3)	−0.3227 (2)	0.2930 (2)	0.0562 (11)	
C12A	0.7683 (3)	−0.3668 (3)	0.2524 (2)	0.0712 (13)	
C13A	0.4820 (3)	−0.3254 (2)	0.4302 (2)	0.0699 (6)	
H13A	0.4200	−0.3289	0.4150	0.084*	0.50
H13B	0.5108	−0.2774	0.4523	0.084*	0.50
H13C	0.4357	−0.3022	0.4196	0.084*	0.50
H13D	0.5286	−0.2932	0.4613	0.084*	0.50
C14A	0.4842 (7)	−0.3832 (4)	0.4756 (3)	0.0699 (6)	0.50
H14A	0.4378	−0.3885	0.5077	0.084*	0.50
H14B	0.5439	−0.3753	0.4979	0.084*	0.50
C15A	0.4615 (5)	−0.4462 (4)	0.4197 (4)	0.0699 (6)	0.50
H15A	0.4009	−0.4541	0.3989	0.084*	0.50
H15B	0.4657	−0.4904	0.4361	0.084*	0.50
C14C	0.4465 (6)	−0.3971 (3)	0.4574 (5)	0.0699 (6)	0.50
H14C	0.3818	−0.4167	0.4446	0.084*	0.50
H14D	0.4533	−0.3896	0.5055	0.084*	0.50
C15C	0.4960 (6)	−0.4519 (5)	0.4327 (4)	0.0699 (6)	0.50
H15C	0.5434	−0.4539	0.4649	0.084*	0.50
H15D	0.4540	−0.4998	0.4218	0.084*	0.50
C16A	0.5360 (3)	−0.4195 (2)	0.3709 (2)	0.0699 (6)	
H16A	0.5948	−0.4241	0.3862	0.084*	0.50
H16B	0.5191	−0.4444	0.3266	0.084*	0.50
H16C	0.6009	−0.4149	0.3752	0.084*	0.50
H16D	0.5108	−0.4495	0.3299	0.084*	0.50
C17A	0.6420 (3)	−0.1824 (2)	0.3977 (2)	0.0794 (14)	
H17A	0.6800	−0.2118	0.4108	0.095*	0.50
H17B	0.6063	−0.1730	0.4343	0.095*	0.50
H17C	0.6979	−0.1963	0.4018	0.095*	0.50
H17D	0.6057	−0.1976	0.4348	0.095*	0.50
C18A	0.6983 (7)	−0.1126 (5)	0.3726 (8)	0.094 (4)	0.50
H18A	0.7432	−0.1211	0.3426	0.113*	0.50
H18B	0.7285	−0.0766	0.4088	0.113*	0.50
C19A	0.6210 (11)	−0.0915 (6)	0.3363 (9)	0.137 (11)	0.50
H19A	0.5777	−0.0808	0.3666	0.164*	0.50
H19B	0.6446	−0.0510	0.3111	0.164*	0.50
C18C	0.6491 (14)	−0.1026 (4)	0.3952 (10)	0.147 (8)	0.50
H18C	0.7072	−0.0729	0.4161	0.176*	0.50
H18D	0.6005	−0.0905	0.4190	0.176*	0.50
C19C	0.6412 (12)	−0.0887 (5)	0.3220 (9)	0.124 (10)	0.50
H19C	0.6131	−0.0504	0.3169	0.149*	0.50
H19D	0.7003	−0.0768	0.3030	0.149*	0.50
C20A	0.5794 (3)	−0.1620 (2)	0.2910 (2)	0.0711 (13)	
H20A	0.5171	−0.1666	0.2755	0.085*	0.50
H20B	0.6155	−0.1656	0.2531	0.085*	0.50
H20C	0.5174	−0.1584	0.2908	0.085*	0.50
H20D	0.5930	−0.1741	0.2457	0.085*	0.50
C21A	0.3572 (3)	−0.3111 (2)	−0.0710 (2)	0.0569 (11)	
C22A	0.2631 (3)	−0.3580 (3)	−0.1026 (2)	0.0715 (13)	
C23A	0.5245 (3)	−0.3923 (2)	−0.0250 (2)	0.0754 (7)	
H23A	0.4952	−0.3969	−0.0690	0.090*	0.50
H23B	0.4840	−0.4251	0.0015	0.090*	0.50
H23C	0.5049	−0.3878	−0.0699	0.090*	0.50
H23D	0.4757	−0.4272	−0.0063	0.090*	0.50
C24A	0.6165 (4)	−0.4090 (4)	−0.0288 (5)	0.0754 (7)	0.50
H24A	0.6225	−0.4415	0.0033	0.090*	0.50
H24B	0.6229	−0.4306	−0.0728	0.090*	0.50
C25A	0.6874 (4)	−0.3355 (4)	−0.0127 (4)	0.0754 (7)	0.50
H25A	0.7026	−0.3122	−0.0527	0.090*	0.50
H25B	0.7428	−0.3406	0.0084	0.090*	0.50
C24C	0.5995 (4)	−0.4228 (4)	0.0008 (5)	0.0754 (7)	0.50
H24C	0.5858	−0.4420	0.0429	0.090*	0.50
H24D	0.6098	−0.4599	−0.0310	0.090*	0.50
C25C	0.6805 (5)	−0.3546 (3)	0.0085 (5)	0.0754 (7)	0.50
H25C	0.7273	−0.3593	0.0399	0.090*	0.50
H25D	0.7070	−0.3461	−0.0338	0.090*	0.50
C26A	0.6426 (3)	−0.2927 (2)	0.0339 (2)	0.0754 (7)	
H26A	0.6477	−0.3042	0.0791	0.090*	0.50
H26B	0.6690	−0.2413	0.0327	0.090*	0.50
H26C	0.6558	−0.2778	0.0810	0.090*	0.50
H26D	0.6645	−0.2507	0.0102	0.090*	0.50
C27A	0.5640 (4)	−0.1587 (2)	−0.0078 (3)	0.0899 (16)	
H27A	0.5456	−0.1834	−0.0521	0.108*	
H27B	0.6289	−0.1513	0.0010	0.108*	
C28A	0.5398 (5)	−0.0873 (3)	0.0007 (4)	0.136 (3)	
H28A	0.5896	−0.0482	−0.0117	0.163*	
H28B	0.4853	−0.0903	−0.0268	0.163*	
C29A	0.5232 (5)	−0.0745 (3)	0.0738 (4)	0.131 (2)	
H29A	0.4819	−0.0450	0.0805	0.157*	
H29B	0.5800	−0.0517	0.1000	0.157*	
C30A	0.4801 (4)	−0.1510 (2)	0.0910 (3)	0.0883 (16)	
H30A	0.5003	−0.1559	0.1357	0.106*	
H30B	0.4140	−0.1623	0.0881	0.106*	
Er1B	−0.082106 (10)	−0.877962 (9)	0.314444 (8)	0.03791 (5)	
Cl21	−0.15835 (7)	−0.93919 (5)	0.41462 (5)	0.0559 (3)	
Cl22	−0.20229 (7)	−0.95434 (6)	0.22130 (5)	0.0628 (3)	
Cl23	0.03315 (7)	−0.95347 (5)	0.29919 (5)	0.0582 (3)	
Cl1B	−0.38356 (11)	−0.94265 (8)	0.50753 (8)	0.1087 (5)	
Cl2B	−0.22102 (8)	−0.84091 (9)	0.56809 (6)	0.0896 (4)	
Cl3B	−0.39434 (11)	−0.82077 (10)	0.59384 (7)	0.1180 (6)	
Cl4B	0.43170 (9)	−0.82764 (12)	0.40212 (9)	0.1353 (7)	
Cl5B	0.27081 (15)	−0.94574 (10)	0.40625 (10)	0.1350 (7)	
Cl6B	0.29476 (10)	−0.86790 (11)	0.29392 (6)	0.1156 (6)	
Cl7B	−0.21453 (12)	−0.97432 (9)	0.03604 (8)	0.1203 (6)	
Cl8B	−0.22648 (13)	−0.87278 (13)	−0.05527 (7)	0.1485 (8)	
Cl9B	−0.29488 (10)	−0.86126 (11)	0.07409 (8)	0.1136 (5)	
P1B	−0.25879 (6)	−0.79124 (5)	0.35098 (5)	0.0417 (2)	
P2B	0.10165 (6)	−0.78402 (5)	0.43520 (5)	0.0386 (2)	
P3B	0.00476 (7)	−0.80148 (6)	0.16537 (5)	0.0485 (3)	
O1B	−0.17322 (16)	−0.80455 (13)	0.32940 (12)	0.0460 (6)	
O2B	−0.37712 (19)	−0.77140 (17)	0.46714 (13)	0.0661 (8)	
O3B	0.02276 (15)	−0.80055 (13)	0.38553 (12)	0.0469 (6)	
O4B	0.3057 (2)	−0.7634 (2)	0.46037 (16)	0.0804 (10)	
O5B	−0.01783 (17)	−0.81319 (14)	0.23410 (12)	0.0522 (7)	
O6B	−0.0624 (2)	−0.7974 (2)	0.02172 (16)	0.0976 (12)	
N1B	−0.26726 (19)	−0.81973 (16)	0.42744 (14)	0.0454 (7)	
H1BA	−0.2299	−0.8429	0.4389	0.055*	
N2B	−0.2496 (2)	−0.70695 (17)	0.35058 (16)	0.0520 (8)	
N3B	−0.3552 (2)	−0.83486 (17)	0.31241 (16)	0.0518 (8)	
N4B	0.17639 (19)	−0.82102 (16)	0.39623 (14)	0.0455 (7)	
H4BC	0.1559	−0.8501	0.3608	0.055*	
N5B	0.0890 (2)	−0.81876 (15)	0.50382 (14)	0.0438 (7)	
N6B	0.1382 (2)	−0.69854 (16)	0.45317 (15)	0.0500 (8)	
N7B	−0.0965 (2)	−0.83826 (18)	0.12142 (15)	0.0544 (8)	
H7BC	−0.1414	−0.8619	0.1423	0.065*	
N8B	0.0720 (3)	−0.8414 (3)	0.1297 (2)	0.1020 (9)	
N9B	0.0457 (3)	−0.7184 (2)	0.1615 (2)	0.0908 (8)	
C1B	−0.3269 (3)	−0.8096 (2)	0.47179 (19)	0.0478 (9)	
C2B	−0.3308 (3)	−0.8520 (2)	0.5337 (2)	0.0597 (11)	
C3B	−0.1608 (3)	−0.6525 (2)	0.3659 (2)	0.0647 (12)	
H3BA	−0.1204	−0.6545	0.3303	0.078*	
H3BB	−0.1319	−0.6601	0.4068	0.078*	
C4B	−0.1826 (3)	−0.5803 (2)	0.3729 (3)	0.0913 (16)	
H4BA	−0.1443	−0.5475	0.4085	0.110*	
H4BB	−0.1736	−0.5579	0.3319	0.110*	
C5B	−0.2823 (3)	−0.5998 (2)	0.3887 (3)	0.0930 (17)	
H5BA	−0.2879	−0.6010	0.4362	0.112*	
H5BB	−0.3109	−0.5648	0.3740	0.112*	
C6B	−0.3270 (3)	−0.6738 (2)	0.3520 (2)	0.0670 (12)	
H6BA	−0.3770	−0.7012	0.3757	0.080*	
H6BB	−0.3495	−0.6704	0.3075	0.080*	
C7B	−0.3796 (4)	−0.8202 (3)	0.2450 (2)	0.0910 (16)	
H7BA	−0.3253	−0.7988	0.2223	0.109*	0.50
H7BB	−0.4180	−0.7880	0.2468	0.109*	0.50
H7DA	−0.3320	−0.8242	0.2157	0.109*	0.50
H7DB	−0.3876	−0.7724	0.2451	0.109*	0.50
C8B	−0.4315 (10)	−0.8949 (6)	0.2106 (8)	0.099 (6)	0.50
H8BA	−0.3899	−0.9170	0.1876	0.118*	0.50
H8BB	−0.4781	−0.8911	0.1785	0.118*	0.50
C9B	−0.4754 (7)	−0.9399 (10)	0.2653 (5)	0.094 (6)	0.50
H9BA	−0.4857	−0.9911	0.2529	0.113*	0.50
H9BB	−0.5325	−0.9308	0.2770	0.113*	0.50
C8D	−0.4658 (6)	−0.8819 (5)	0.2229 (8)	0.076 (4)	0.50
H8DA	−0.4751	−0.8902	0.1750	0.092*	0.50
H8DB	−0.5196	−0.8728	0.2415	0.092*	0.50
C9D	−0.4428 (9)	−0.9454 (7)	0.2518 (4)	0.079 (5)	0.50
H9DA	−0.3996	−0.9625	0.2257	0.095*	0.50
H9DB	−0.4972	−0.9851	0.2547	0.095*	0.50
C10B	−0.3999 (3)	−0.9101 (2)	0.3213 (2)	0.0669 (12)	
H10E	−0.4262	−0.9132	0.3642	0.080*	0.50
H10F	−0.3560	−0.9377	0.3192	0.080*	0.50
H10K	−0.4451	−0.9137	0.3543	0.080*	0.50
H10L	−0.3556	−0.9340	0.3348	0.080*	0.50
C11B	0.2657 (3)	−0.8090 (3)	0.4157 (2)	0.0577 (11)	
C12B	0.3147 (3)	−0.8598 (3)	0.3794 (2)	0.0775 (14)	
C13B	0.0368 (3)	−0.7952 (2)	0.5564 (2)	0.0715 (13)	
H13E	−0.0257	−0.8010	0.5406	0.086*	0.50
H13F	0.0651	−0.7453	0.5738	0.086*	0.50
H13K	−0.0138	−0.7814	0.5370	0.086*	0.50
H13L	0.0751	−0.7542	0.5850	0.086*	0.50
C14B	0.0412 (8)	−0.8465 (5)	0.6089 (4)	0.055 (3)	0.50
H14E	0.1009	−0.8341	0.6322	0.067*	0.50
H14F	−0.0054	−0.8484	0.6406	0.067*	0.50
C15B	0.0216 (8)	−0.9166 (5)	0.5619 (5)	0.068 (4)	0.50
H15E	−0.0403	−0.9309	0.5418	0.082*	0.50
H15F	0.0316	−0.9558	0.5845	0.082*	0.50
C14D	−0.0029 (9)	−0.8625 (7)	0.5919 (8)	0.110 (7)	0.50
H14G	−0.0037	−0.8492	0.6392	0.133*	0.50
H14H	−0.0650	−0.8871	0.5746	0.133*	0.50
C15D	0.0590 (12)	−0.9116 (12)	0.5791 (6)	0.132 (10)	0.50
H15G	0.0250	−0.9621	0.5778	0.158*	0.50
H15H	0.1094	−0.9003	0.6125	0.158*	0.50
C16B	0.0929 (3)	−0.8930 (2)	0.5105 (2)	0.0738 (13)	
H16E	0.1532	−0.8934	0.5267	0.089*	0.50
H16F	0.0760	−0.9236	0.4687	0.089*	0.50
H16K	0.1559	−0.8943	0.5115	0.089*	0.50
H16L	0.0594	−0.9259	0.4732	0.089*	0.50
C17B	0.2007 (3)	−0.6585 (2)	0.5108 (2)	0.0678 (12)	
H17E	0.2482	−0.6813	0.5195	0.081*	0.50
H17F	0.1673	−0.6561	0.5504	0.081*	0.50
H17K	0.2338	−0.6883	0.5303	0.081*	0.50
H17L	0.1695	−0.6379	0.5448	0.081*	0.50
C18B	0.2415 (15)	−0.5831 (5)	0.4891 (9)	0.133 (10)	0.50
H18E	0.2249	−0.5480	0.5196	0.159*	0.50
H18F	0.3076	−0.5718	0.4921	0.159*	0.50
C19B	0.2095 (12)	−0.5759 (7)	0.4182 (8)	0.096 (7)	0.50
H19E	0.1825	−0.5364	0.4170	0.115*	0.50
H19F	0.2591	−0.5693	0.3886	0.115*	0.50
C18D	0.2647 (8)	−0.5967 (5)	0.4783 (6)	0.063 (4)	0.50
H18G	0.3047	−0.6132	0.4485	0.075*	0.50
H18H	0.3001	−0.5579	0.5104	0.075*	0.50
C19D	0.1872 (9)	−0.5769 (4)	0.4412 (6)	0.075 (5)	0.50
H19G	0.1474	−0.5613	0.4718	0.091*	0.50
H19H	0.2110	−0.5395	0.4126	0.091*	0.50
C20B	0.1379 (3)	−0.6493 (2)	0.4008 (2)	0.0761 (14)	
H20E	0.0778	−0.6422	0.3959	0.091*	0.50
H20F	0.1504	−0.6716	0.3587	0.091*	0.50
H20K	0.0758	−0.6516	0.3865	0.091*	0.50
H20L	0.1698	−0.6604	0.3625	0.091*	0.50
C21B	−0.1128 (4)	−0.8336 (3)	0.0561 (2)	0.0690 (13)	
C22B	−0.2049 (4)	−0.8808 (3)	0.0298 (2)	0.0808 (15)	
C23B	0.0432 (4)	−0.9182 (3)	0.1039 (3)	0.1020 (9)	
H23E	0.0176	−0.9231	0.0587	0.122*	0.50
H23F	−0.0047	−0.9438	0.1303	0.122*	0.50
H23K	0.0327	−0.9246	0.0560	0.122*	0.50
H23L	−0.0130	−0.9424	0.1231	0.122*	0.50
C24B	0.1204 (6)	−0.9534 (6)	0.1046 (6)	0.1020 (9)	0.50
H24E	0.1304	−0.9721	0.0604	0.122*	0.50
H24H	0.1090	−0.9919	0.1332	0.122*	0.50
C25B	0.2012 (8)	−0.8887 (4)	0.1330 (6)	0.1020 (9)	0.50
H25E	0.2477	−0.8799	0.1009	0.122*	0.50
H25F	0.2277	−0.9004	0.1730	0.122*	0.50
C24D	0.1323 (5)	−0.9328 (6)	0.1307 (6)	0.1020 (9)	0.50
H24F	0.1349	−0.9306	0.1788	0.122*	0.50
H24G	0.1357	−0.9798	0.1124	0.122*	0.50
C25D	0.2102 (8)	−0.8722 (5)	0.1076 (6)	0.1020 (9)	0.50
H25G	0.2709	−0.8726	0.1229	0.122*	0.50
H25H	0.2065	−0.8684	0.0603	0.122*	0.50
C26B	0.1721 (4)	−0.8193 (3)	0.1494 (3)	0.1020 (9)	
H26E	0.1832	−0.8026	0.1964	0.122*	0.50
H26F	0.2051	−0.7811	0.1243	0.122*	0.50
H26K	0.1844	−0.8147	0.1971	0.122*	0.50
H26L	0.2033	−0.7742	0.1328	0.122*	0.50
C27B	0.0986 (4)	−0.6803 (2)	0.1079 (3)	0.0908 (8)	
H27C	0.0844	−0.7091	0.0651	0.109*	0.50
H27D	0.1640	−0.6664	0.1186	0.109*	0.50
H27E	0.0661	−0.6988	0.0651	0.109*	0.50
H27F	0.1587	−0.6877	0.1070	0.109*	0.50
C28B	0.0605 (7)	−0.6143 (4)	0.1107 (4)	0.0908 (8)	0.50
H28C	0.0946	−0.5770	0.0853	0.109*	0.50
H28D	−0.0040	−0.6274	0.0970	0.109*	0.50
C29B	0.0791 (7)	−0.5934 (4)	0.1863 (4)	0.0908 (8)	0.50
H29C	0.1434	−0.5829	0.2000	0.109*	0.50
H29D	0.0569	−0.5530	0.2017	0.109*	0.50
C28D	0.1026 (7)	−0.6021 (3)	0.1281 (5)	0.0908 (8)	0.50
H28E	0.0877	−0.5813	0.0891	0.109*	0.50
H28F	0.1647	−0.5758	0.1439	0.109*	0.50
C29D	0.0380 (7)	−0.5913 (4)	0.1822 (5)	0.0908 (8)	0.50
H29E	−0.0179	−0.5844	0.1637	0.109*	0.50
H29F	0.0673	−0.5514	0.2155	0.109*	0.50
C30B	0.0216 (4)	−0.6649 (2)	0.2093 (3)	0.0908 (8)	
H30C	0.0392	−0.6695	0.2547	0.109*	0.50
H30D	−0.0433	−0.6692	0.2055	0.109*	0.50
H30E	0.0534	−0.6617	0.2522	0.109*	0.50
H30F	−0.0436	−0.6804	0.2149	0.109*	0.50

### Atomic displacement parameters (Å^2^)

**Table:**

	*U*^11^	*U*^22^	*U*^33^	*U*^12^	*U*^13^	*U*^23^
Er1A	0.03493 (9)	0.03913 (10)	0.04077 (10)	0.01110 (7)	0.00295 (7)	0.00414 (8)
Cl11	0.0665 (7)	0.0585 (6)	0.0720 (7)	0.0231 (5)	0.0237 (6)	0.0299 (6)
Cl12	0.0528 (6)	0.0575 (6)	0.0565 (6)	−0.0060 (5)	−0.0077 (5)	0.0032 (5)
Cl13	0.0582 (6)	0.0589 (6)	0.0742 (7)	0.0309 (5)	−0.0055 (5)	−0.0173 (5)
Cl1A	0.0913 (9)	0.0697 (8)	0.1047 (10)	0.0008 (7)	0.0056 (8)	0.0277 (8)
Cl2A	0.1163 (11)	0.1591 (15)	0.0909 (9)	0.0862 (11)	0.0635 (9)	0.0626 (10)
Cl3A	0.0664 (7)	0.1135 (10)	0.0661 (7)	0.0300 (7)	−0.0062 (6)	0.0308 (7)
Cl4A	0.0635 (9)	0.287 (3)	0.1412 (15)	0.0879 (13)	−0.0268 (9)	−0.0798 (16)
Cl5A	0.227 (2)	0.1276 (14)	0.1267 (14)	0.1239 (16)	0.0589 (14)	0.0408 (12)
Cl6A	0.0843 (9)	0.1402 (13)	0.0645 (7)	0.0566 (9)	0.0189 (7)	0.0057 (8)
Cl7A	0.1510 (15)	0.0890 (11)	0.1244 (13)	0.0128 (10)	−0.0296 (11)	−0.0291 (10)
Cl8A	0.0641 (8)	0.1925 (17)	0.0753 (9)	0.0369 (10)	0.0016 (7)	0.0006 (10)
Cl9A	0.1154 (12)	0.1982 (19)	0.0553 (8)	0.0033 (12)	−0.0195 (8)	0.0346 (10)
P1A	0.0385 (5)	0.0409 (5)	0.0478 (6)	0.0168 (4)	0.0076 (4)	0.0095 (5)
P2A	0.0386 (5)	0.0388 (5)	0.0391 (5)	0.0113 (4)	−0.0004 (4)	0.0001 (4)
P3A	0.0452 (6)	0.0443 (6)	0.0440 (6)	0.0074 (4)	0.0082 (5)	0.0088 (5)
O1A	0.0482 (15)	0.0488 (15)	0.0524 (15)	0.0199 (12)	0.0124 (12)	0.0120 (12)
O2A	0.0518 (16)	0.080 (2)	0.0585 (17)	0.0371 (15)	0.0132 (14)	0.0144 (15)
O3A	0.0428 (14)	0.0469 (14)	0.0462 (14)	0.0169 (12)	−0.0001 (12)	−0.0047 (12)
O4A	0.0517 (18)	0.123 (3)	0.067 (2)	0.0295 (18)	−0.0164 (16)	−0.025 (2)
O5A	0.0440 (14)	0.0482 (15)	0.0484 (15)	−0.0005 (12)	0.0006 (12)	0.0100 (12)
O6A	0.076 (2)	0.103 (3)	0.0556 (18)	0.0102 (19)	0.0099 (16)	0.0314 (19)
N1A	0.0408 (17)	0.0542 (19)	0.0473 (18)	0.0228 (15)	0.0058 (14)	0.0123 (15)
N2A	0.0464 (19)	0.0423 (18)	0.078 (2)	0.0208 (15)	0.0132 (17)	0.0127 (17)
N3A	0.053 (2)	0.052 (2)	0.0479 (18)	0.0180 (16)	−0.0007 (16)	0.0108 (16)
N4A	0.0386 (17)	0.058 (2)	0.0428 (17)	0.0180 (15)	−0.0042 (14)	−0.0028 (15)
N5A	0.0774 (16)	0.0723 (14)	0.0679 (14)	0.0288 (13)	0.0168 (12)	0.0207 (12)
N6A	0.061 (2)	0.0439 (19)	0.0515 (19)	0.0121 (16)	−0.0046 (17)	0.0001 (16)
N7A	0.0459 (18)	0.055 (2)	0.0455 (18)	0.0056 (15)	0.0064 (15)	0.0105 (16)
N8A	0.0590 (13)	0.0760 (15)	0.0900 (18)	0.0204 (12)	0.0098 (12)	−0.0012 (13)
N9A	0.069 (2)	0.0415 (19)	0.071 (2)	0.0079 (17)	0.0212 (19)	0.0130 (18)
C1A	0.036 (2)	0.066 (3)	0.042 (2)	0.0164 (19)	0.0060 (17)	0.009 (2)
C2A	0.049 (2)	0.068 (3)	0.061 (3)	0.026 (2)	0.016 (2)	0.023 (2)
C3A	0.059 (3)	0.051 (3)	0.080 (3)	0.009 (2)	0.006 (2)	0.007 (2)
C4A	0.115 (14)	0.074 (11)	0.079 (12)	0.016 (9)	0.013 (10)	−0.007 (7)
C5A	0.126 (12)	0.074 (9)	0.068 (8)	0.060 (8)	−0.005 (9)	−0.007 (6)
C4C	0.079 (9)	0.027 (6)	0.077 (11)	0.011 (5)	0.035 (8)	0.010 (5)
C5C	0.116 (11)	0.056 (7)	0.080 (9)	0.060 (7)	0.005 (9)	−0.011 (6)
C6A	0.072 (3)	0.064 (3)	0.130 (5)	0.043 (3)	0.029 (3)	0.022 (3)
C7A	0.100 (4)	0.102 (4)	0.062 (3)	0.032 (3)	−0.006 (3)	0.019 (3)
C8A	0.048 (7)	0.139 (13)	0.044 (6)	0.018 (8)	−0.011 (5)	0.017 (7)
C9A	0.032 (7)	0.070 (8)	0.115 (11)	−0.021 (7)	−0.013 (6)	0.008 (8)
C8C	0.079 (13)	0.19 (3)	0.129 (17)	−0.040 (13)	−0.023 (11)	0.010 (14)
C9C	0.041 (8)	0.107 (11)	0.119 (11)	0.006 (9)	−0.020 (7)	0.001 (9)
C10A	0.058 (3)	0.058 (3)	0.080 (3)	0.008 (2)	−0.008 (2)	0.002 (2)
C11A	0.046 (2)	0.072 (3)	0.053 (3)	0.022 (2)	0.002 (2)	0.005 (2)
C12A	0.060 (3)	0.105 (4)	0.060 (3)	0.047 (3)	−0.002 (2)	−0.001 (3)
C13A	0.0774 (16)	0.0723 (14)	0.0679 (14)	0.0288 (13)	0.0168 (12)	0.0207 (12)
C14A	0.0774 (16)	0.0723 (14)	0.0679 (14)	0.0288 (13)	0.0168 (12)	0.0207 (12)
C15A	0.0774 (16)	0.0723 (14)	0.0679 (14)	0.0288 (13)	0.0168 (12)	0.0207 (12)
C14C	0.0774 (16)	0.0723 (14)	0.0679 (14)	0.0288 (13)	0.0168 (12)	0.0207 (12)
C15C	0.0774 (16)	0.0723 (14)	0.0679 (14)	0.0288 (13)	0.0168 (12)	0.0207 (12)
C16A	0.0774 (16)	0.0723 (14)	0.0679 (14)	0.0288 (13)	0.0168 (12)	0.0207 (12)
C17A	0.086 (3)	0.065 (3)	0.075 (3)	0.016 (3)	−0.028 (3)	−0.023 (3)
C18A	0.069 (8)	0.034 (5)	0.161 (14)	−0.006 (5)	−0.027 (8)	−0.012 (7)
C19A	0.173 (18)	0.059 (11)	0.121 (13)	−0.063 (11)	−0.076 (12)	0.054 (10)
C18C	0.124 (15)	0.093 (11)	0.196 (19)	0.023 (10)	−0.014 (14)	−0.086 (12)
C19C	0.153 (17)	0.043 (10)	0.17 (2)	0.032 (11)	−0.021 (15)	−0.001 (11)
C20A	0.093 (4)	0.045 (3)	0.077 (3)	0.019 (2)	0.005 (3)	0.017 (2)
C21A	0.060 (3)	0.065 (3)	0.047 (2)	0.018 (2)	0.009 (2)	0.006 (2)
C22A	0.073 (3)	0.083 (3)	0.050 (3)	0.012 (3)	−0.001 (2)	0.001 (2)
C23A	0.0590 (13)	0.0760 (15)	0.0900 (18)	0.0204 (12)	0.0098 (12)	−0.0012 (13)
C24A	0.0590 (13)	0.0760 (15)	0.0900 (18)	0.0204 (12)	0.0098 (12)	−0.0012 (13)
C25A	0.0590 (13)	0.0760 (15)	0.0900 (18)	0.0204 (12)	0.0098 (12)	−0.0012 (13)
C24C	0.0590 (13)	0.0760 (15)	0.0900 (18)	0.0204 (12)	0.0098 (12)	−0.0012 (13)
C25C	0.0590 (13)	0.0760 (15)	0.0900 (18)	0.0204 (12)	0.0098 (12)	−0.0012 (13)
C26A	0.0590 (13)	0.0760 (15)	0.0900 (18)	0.0204 (12)	0.0098 (12)	−0.0012 (13)
C27A	0.099 (4)	0.066 (3)	0.093 (4)	−0.007 (3)	0.023 (3)	0.040 (3)
C28A	0.131 (6)	0.071 (4)	0.195 (8)	−0.003 (4)	−0.006 (6)	0.067 (5)
C29A	0.182 (7)	0.067 (4)	0.145 (6)	0.038 (4)	0.032 (6)	0.011 (4)
C30A	0.102 (4)	0.058 (3)	0.103 (4)	0.020 (3)	0.023 (3)	0.009 (3)
Er1B	0.03557 (9)	0.03848 (9)	0.04025 (10)	0.01111 (7)	0.00430 (7)	0.00495 (7)
Cl21	0.0579 (6)	0.0553 (6)	0.0626 (6)	0.0215 (5)	0.0184 (5)	0.0248 (5)
Cl22	0.0583 (6)	0.0592 (6)	0.0568 (6)	−0.0027 (5)	−0.0045 (5)	−0.0006 (5)
Cl23	0.0587 (6)	0.0539 (6)	0.0692 (7)	0.0295 (5)	0.0078 (5)	−0.0006 (5)
Cl1B	0.1153 (12)	0.0744 (9)	0.1195 (12)	−0.0072 (8)	−0.0037 (9)	0.0372 (9)
Cl2B	0.0629 (7)	0.1514 (13)	0.0606 (7)	0.0352 (8)	−0.0020 (6)	0.0305 (8)
Cl3B	0.1374 (13)	0.1765 (17)	0.0880 (10)	0.1005 (13)	0.0676 (10)	0.0607 (11)
Cl4B	0.0568 (8)	0.218 (2)	0.1351 (13)	0.0699 (11)	−0.0142 (8)	−0.0493 (13)
Cl5B	0.1744 (17)	0.1171 (14)	0.1502 (16)	0.0996 (13)	0.0283 (13)	0.0134 (12)
Cl6B	0.0957 (10)	0.2123 (19)	0.0612 (8)	0.0880 (12)	0.0090 (7)	−0.0101 (10)
Cl7B	0.1353 (14)	0.0991 (12)	0.1079 (12)	0.0173 (10)	−0.0181 (10)	−0.0223 (10)
Cl8B	0.1341 (15)	0.237 (2)	0.0577 (8)	0.0241 (15)	−0.0219 (9)	0.0310 (11)
Cl9B	0.0736 (9)	0.1709 (16)	0.0948 (10)	0.0433 (10)	−0.0034 (8)	−0.0153 (11)
P1B	0.0392 (5)	0.0434 (5)	0.0467 (6)	0.0180 (4)	0.0048 (4)	0.0068 (5)
P2B	0.0341 (5)	0.0386 (5)	0.0427 (5)	0.0112 (4)	0.0000 (4)	0.0008 (4)
P3B	0.0507 (6)	0.0504 (6)	0.0468 (6)	0.0139 (5)	0.0121 (5)	0.0155 (5)
O1B	0.0469 (15)	0.0469 (15)	0.0495 (15)	0.0196 (12)	0.0058 (12)	0.0120 (12)
O2B	0.0611 (18)	0.094 (2)	0.0603 (18)	0.0467 (17)	0.0107 (15)	0.0126 (16)
O3B	0.0382 (14)	0.0443 (14)	0.0584 (16)	0.0151 (11)	0.0014 (12)	−0.0021 (12)
O4B	0.0490 (18)	0.112 (3)	0.076 (2)	0.0287 (18)	−0.0116 (16)	−0.021 (2)
O5B	0.0521 (16)	0.0521 (16)	0.0479 (15)	0.0043 (13)	0.0022 (13)	0.0158 (13)
O6B	0.092 (3)	0.136 (3)	0.061 (2)	0.015 (2)	0.0151 (19)	0.041 (2)
N1B	0.0408 (17)	0.057 (2)	0.0471 (18)	0.0256 (15)	0.0062 (14)	0.0096 (15)
N2B	0.0458 (18)	0.0474 (19)	0.068 (2)	0.0203 (16)	0.0058 (16)	0.0096 (17)
N3B	0.0463 (19)	0.051 (2)	0.058 (2)	0.0154 (16)	−0.0074 (16)	0.0038 (17)
N4B	0.0369 (17)	0.058 (2)	0.0428 (17)	0.0173 (15)	0.0011 (14)	−0.0014 (15)
N5B	0.0489 (18)	0.0402 (17)	0.0460 (17)	0.0178 (14)	0.0115 (14)	0.0042 (14)
N6B	0.055 (2)	0.0389 (17)	0.0503 (19)	0.0060 (15)	−0.0092 (16)	0.0034 (15)
N7B	0.055 (2)	0.064 (2)	0.0425 (18)	0.0127 (17)	0.0054 (16)	0.0120 (17)
N8B	0.0985 (19)	0.123 (2)	0.101 (2)	0.0570 (19)	0.0265 (18)	0.0085 (19)
N9B	0.096 (2)	0.0698 (16)	0.0987 (19)	0.0036 (15)	0.0124 (15)	0.0283 (14)
C1B	0.039 (2)	0.056 (2)	0.046 (2)	0.0120 (19)	−0.0018 (18)	0.0012 (19)
C2B	0.059 (3)	0.068 (3)	0.060 (3)	0.026 (2)	0.016 (2)	0.015 (2)
C3B	0.050 (3)	0.056 (3)	0.084 (3)	0.009 (2)	−0.005 (2)	0.003 (2)
C4B	0.096 (4)	0.050 (3)	0.122 (5)	0.013 (3)	0.010 (3)	0.003 (3)
C5B	0.099 (4)	0.065 (3)	0.124 (5)	0.043 (3)	0.010 (4)	−0.007 (3)
C6B	0.069 (3)	0.062 (3)	0.087 (3)	0.041 (2)	0.016 (3)	0.019 (3)
C7B	0.096 (4)	0.107 (4)	0.065 (3)	0.022 (3)	−0.027 (3)	0.012 (3)
C8B	0.057 (9)	0.146 (16)	0.072 (10)	0.001 (9)	−0.012 (8)	−0.001 (9)
C9B	0.040 (8)	0.109 (12)	0.110 (11)	0.006 (8)	−0.018 (7)	−0.045 (10)
C8D	0.047 (7)	0.122 (11)	0.059 (7)	0.026 (8)	−0.007 (6)	0.000 (7)
C9D	0.031 (6)	0.098 (10)	0.096 (11)	0.001 (7)	−0.009 (6)	0.009 (8)
C10B	0.051 (3)	0.068 (3)	0.076 (3)	0.012 (2)	−0.004 (2)	−0.002 (3)
C11B	0.046 (3)	0.073 (3)	0.056 (3)	0.021 (2)	0.005 (2)	0.008 (2)
C12B	0.061 (3)	0.115 (4)	0.068 (3)	0.048 (3)	0.002 (2)	−0.007 (3)
C13B	0.086 (3)	0.064 (3)	0.081 (3)	0.041 (3)	0.040 (3)	0.019 (3)
C14B	0.055 (7)	0.064 (6)	0.052 (6)	0.023 (5)	0.020 (5)	0.007 (5)
C15B	0.095 (10)	0.052 (6)	0.081 (7)	0.049 (6)	0.026 (7)	0.032 (5)
C14D	0.104 (13)	0.165 (18)	0.090 (11)	0.062 (11)	0.037 (10)	0.071 (12)
C15D	0.135 (15)	0.193 (19)	0.135 (15)	0.115 (14)	0.093 (13)	0.116 (14)
C16B	0.100 (4)	0.058 (3)	0.083 (3)	0.045 (3)	0.028 (3)	0.027 (3)
C17B	0.070 (3)	0.052 (3)	0.074 (3)	0.014 (2)	−0.019 (2)	−0.008 (2)
C18B	0.142 (18)	0.039 (8)	0.21 (2)	0.010 (9)	0.028 (15)	0.001 (10)
C19B	0.103 (12)	0.082 (10)	0.091 (11)	−0.006 (7)	0.014 (9)	0.046 (8)
C18D	0.056 (6)	0.028 (5)	0.091 (8)	−0.003 (5)	−0.025 (6)	−0.002 (5)
C19D	0.088 (10)	0.052 (7)	0.078 (9)	−0.003 (6)	−0.010 (8)	0.045 (6)
C20B	0.079 (3)	0.060 (3)	0.087 (3)	0.012 (3)	−0.016 (3)	0.027 (3)
C21B	0.074 (3)	0.085 (4)	0.049 (3)	0.026 (3)	0.000 (3)	0.010 (3)
C22B	0.116 (4)	0.076 (3)	0.046 (3)	0.018 (3)	0.027 (3)	0.001 (2)
C23B	0.0985 (19)	0.123 (2)	0.101 (2)	0.0570 (19)	0.0265 (18)	0.0085 (19)
C24B	0.0985 (19)	0.123 (2)	0.101 (2)	0.0570 (19)	0.0265 (18)	0.0085 (19)
C25B	0.0985 (19)	0.123 (2)	0.101 (2)	0.0570 (19)	0.0265 (18)	0.0085 (19)
C24D	0.0985 (19)	0.123 (2)	0.101 (2)	0.0570 (19)	0.0265 (18)	0.0085 (19)
C25D	0.0985 (19)	0.123 (2)	0.101 (2)	0.0570 (19)	0.0265 (18)	0.0085 (19)
C26B	0.0985 (19)	0.123 (2)	0.101 (2)	0.0570 (19)	0.0265 (18)	0.0085 (19)
C27B	0.096 (2)	0.0698 (16)	0.0987 (19)	0.0036 (15)	0.0124 (15)	0.0283 (14)
C28B	0.096 (2)	0.0698 (16)	0.0987 (19)	0.0036 (15)	0.0124 (15)	0.0283 (14)
C29B	0.096 (2)	0.0698 (16)	0.0987 (19)	0.0036 (15)	0.0124 (15)	0.0283 (14)
C28D	0.096 (2)	0.0698 (16)	0.0987 (19)	0.0036 (15)	0.0124 (15)	0.0283 (14)
C29D	0.096 (2)	0.0698 (16)	0.0987 (19)	0.0036 (15)	0.0124 (15)	0.0283 (14)
C30B	0.096 (2)	0.0698 (16)	0.0987 (19)	0.0036 (15)	0.0124 (15)	0.0283 (14)

### Geometric parameters (Å, °)

**Table:**

Er1A—O3A	2.229 (2)	Er1B—O3B	2.232 (2)
Er1A—O5A	2.247 (2)	Er1B—O5B	2.238 (2)
Er1A—O1A	2.258 (2)	Er1B—O1B	2.267 (2)
Er1A—Cl12	2.5938 (11)	Er1B—Cl21	2.5963 (10)
Er1A—Cl13	2.6036 (11)	Er1B—Cl23	2.6012 (11)
Er1A—Cl11	2.6078 (11)	Er1B—Cl22	2.6153 (11)
Cl1A—C2A	1.772 (5)	Cl1B—C2B	1.740 (5)
Cl2A—C2A	1.755 (4)	Cl2B—C2B	1.743 (4)
Cl3A—C2A	1.769 (4)	Cl3B—C2B	1.753 (4)
Cl4A—C12A	1.724 (5)	Cl4B—C12B	1.749 (5)
Cl5A—C12A	1.785 (6)	Cl5B—C12B	1.761 (6)
Cl6A—C12A	1.754 (5)	Cl6B—C12B	1.735 (5)
Cl7A—C22A	1.782 (5)	Cl7B—C22B	1.799 (5)
Cl8A—C22A	1.743 (5)	Cl8B—C22B	1.775 (5)
Cl9A—C22A	1.730 (5)	Cl9B—C22B	1.777 (5)
P1A—O1A	1.481 (2)	P1B—O1B	1.476 (2)
P1A—N2A	1.604 (3)	P1B—N2B	1.606 (3)
P1A—N3A	1.613 (3)	P1B—N3B	1.610 (3)
P1A—N1A	1.693 (3)	P1B—N1B	1.693 (3)
P2A—O3A	1.480 (2)	P2B—O3B	1.486 (3)
P2A—N5A	1.603 (4)	P2B—N5B	1.598 (3)
P2A—N6A	1.605 (3)	P2B—N6B	1.598 (3)
P2A—N4A	1.685 (3)	P2B—N4B	1.692 (3)
P3A—O5A	1.480 (2)	P3B—O5B	1.471 (3)
P3A—N8A	1.595 (4)	P3B—N9B	1.569 (4)
P3A—N9A	1.602 (3)	P3B—N8B	1.605 (4)
P3A—N7A	1.687 (3)	P3B—N7B	1.692 (3)
O2A—C1A	1.201 (4)	O2B—C1B	1.216 (4)
O4A—C11A	1.191 (5)	O4B—C11B	1.218 (5)
O6A—C21A	1.196 (5)	O6B—C21B	1.184 (5)
N1A—C1A	1.354 (4)	N1B—C1B	1.342 (4)
N1A—H1AA	0.8600	N1B—H1BA	0.8600
N2A—C3A	1.457 (5)	N2B—C3B	1.469 (5)
N2A—C6A	1.487 (5)	N2B—C6B	1.497 (5)
N3A—C10A	1.468 (5)	N3B—C10B	1.460 (5)
N3A—C7A	1.491 (5)	N3B—C7B	1.479 (5)
N4A—C11A	1.358 (5)	N4B—C11B	1.356 (5)
N4A—H4AC	0.8600	N4B—H4BC	0.8600
N5A—C16A	1.481 (5)	N5B—C13B	1.469 (5)
N5A—C13A	1.488 (5)	N5B—C16B	1.480 (5)
N6A—C20A	1.474 (5)	N6B—C17B	1.489 (5)
N6A—C17A	1.488 (5)	N6B—C20B	1.497 (5)
N7A—C21A	1.366 (5)	N7B—C21B	1.355 (5)
N7A—H7AC	0.8600	N7B—H7BC	0.8600
N8A—C26A	1.485 (5)	N8B—C23B	1.471 (7)
N8A—C23A	1.484 (6)	N8B—C26B	1.497 (7)
N9A—C30A	1.455 (6)	N9B—C30B	1.487 (7)
N9A—C27A	1.483 (5)	N9B—C27B	1.503 (6)
C1A—C2A	1.548 (5)	C1B—C2B	1.560 (5)
C3A—C4C	1.535 (5)	C3B—C4B	1.528 (4)
C3A—C4A	1.538 (5)	C3B—H3BA	0.9700
C3A—H3AA	0.9700	C3B—H3BB	0.9700
C3A—H3AB	0.9700	C4B—C5B	1.517 (4)
C3A—H3CA	0.9699	C4B—H4BA	0.9700
C3A—H3CB	0.9702	C4B—H4BB	0.9700
C4A—C5A	1.540 (5)	C5B—C6B	1.516 (4)
C4A—H4AA	0.9700	C5B—H5BA	0.9700
C4A—H4AB	0.9700	C5B—H5BB	0.9700
C5A—C6A	1.528 (5)	C6B—H6BA	0.9700
C5A—H5AA	0.9700	C6B—H6BB	0.9700
C5A—H5AB	0.9700	C7B—C8D	1.531 (5)
C4C—C5C	1.538 (5)	C7B—C8B	1.532 (5)
C4C—H4CA	0.9700	C7B—H7BA	0.9700
C4C—H4CB	0.9700	C7B—H7BB	0.9700
C5C—C6A	1.529 (5)	C7B—H7DA	0.9700
C5C—H5CA	0.9700	C7B—H7DB	0.9699
C5C—H5CB	0.9700	C8B—C9B	1.533 (5)
C6A—H6AA	0.9700	C8B—H8BA	0.9700
C6A—H6AB	0.9700	C8B—H8BB	0.9700
C6A—H6CA	0.9702	C9B—C10B	1.539 (5)
C6A—H6CB	0.9700	C9B—H9BA	0.9700
C7A—C8A	1.537 (5)	C9B—H9BB	0.9700
C7A—C8C	1.540 (5)	C8D—C9D	1.535 (5)
C7A—H7AA	0.9700	C8D—H8DA	0.9700
C7A—H7AB	0.9700	C8D—H8DB	0.9700
C7A—H7CA	0.9701	C9D—C10B	1.546 (5)
C7A—H7CB	0.9701	C9D—H9DA	0.9700
C8A—C9A	1.536 (5)	C9D—H9DB	0.9700
C8A—H8AA	0.9700	C10B—H10E	0.9700
C8A—H8AB	0.9700	C10B—H10F	0.9700
C9A—C10A	1.534 (5)	C10B—H10K	0.9700
C9A—H9AA	0.9700	C10B—H10L	0.9699
C9A—H9AB	0.9700	C11B—C12B	1.549 (6)
C8C—C9C	1.535 (5)	C13B—C14D	1.536 (5)
C8C—H8CA	0.9700	C13B—C14B	1.538 (5)
C8C—H8CB	0.9700	C13B—H13E	0.9700
C9C—C10A	1.529 (5)	C13B—H13F	0.9700
C9C—H9CA	0.9700	C13B—H13K	0.9701
C9C—H9CB	0.9700	C13B—H13L	0.9699
C10A—H10A	0.9700	C14B—C15B	1.536 (5)
C10A—H10B	0.9700	C14B—H14E	0.9700
C10A—H10C	0.9700	C14B—H14F	0.9700
C10A—H10D	0.9699	C15B—C16B	1.540 (5)
C11A—C12A	1.554 (6)	C15B—H15E	0.9700
C13A—C14C	1.510 (5)	C15B—H15F	0.9700
C13A—C14A	1.524 (5)	C14D—C15D	1.535 (5)
C13A—H13A	0.9700	C14D—H14G	0.9700
C13A—H13B	0.9700	C14D—H14H	0.9700
C13A—H13C	0.9701	C15D—C16B	1.539 (5)
C13A—H13D	0.9696	C15D—H15G	0.9700
C14A—C15A	1.542 (5)	C15D—H15H	0.9700
C14A—H14A	0.9700	C16B—H16E	0.9700
C14A—H14B	0.9700	C16B—H16F	0.9700
C15A—C16A	1.543 (5)	C16B—H16K	0.9700
C15A—H15A	0.9700	C16B—H16L	0.9700
C15A—H15B	0.9700	C17B—C18B	1.536 (5)
C14C—C15C	1.529 (5)	C17B—C18D	1.538 (5)
C14C—H14C	0.9700	C17B—H17E	0.9700
C14C—H14D	0.9700	C17B—H17F	0.9700
C15C—C16A	1.525 (5)	C17B—H17K	0.9701
C15C—H15C	0.9700	C17B—H17L	0.9701
C15C—H15D	0.9700	C18B—C19B	1.536 (5)
C16A—H16A	0.9700	C18B—H18E	0.9700
C16A—H16B	0.9700	C18B—H18F	0.9700
C16A—H16C	0.9701	C19B—C20B	1.534 (5)
C16A—H16D	0.9700	C19B—H19E	0.9700
C17A—C18C	1.530 (5)	C19B—H19F	0.9700
C17A—C18A	1.531 (5)	C18D—C19D	1.538 (5)
C17A—H17A	0.9700	C18D—H18G	0.9700
C17A—H17B	0.9700	C18D—H18H	0.9700
C17A—H17C	0.9701	C19D—C20B	1.537 (5)
C17A—H17D	0.9698	C19D—H19G	0.9700
C18A—C19A	1.542 (5)	C19D—H19H	0.9700
C18A—H18A	0.9700	C20B—H20E	0.9700
C18A—H18B	0.9700	C20B—H20F	0.9700
C19A—C20A	1.534 (5)	C20B—H20K	0.9699
C19A—H19A	0.9700	C20B—H20L	0.9700
C19A—H19B	0.9700	C21B—C22B	1.495 (7)
C18C—C19C	1.542 (5)	C23B—C24B	1.522 (5)
C18C—H18C	0.9700	C23B—C24D	1.558 (5)
C18C—H18D	0.9700	C23B—H23E	0.9700
C19C—C20A	1.532 (5)	C23B—H23F	0.9700
C19C—H19C	0.9700	C23B—H23K	0.9702
C19C—H19D	0.9700	C23B—H23L	0.9700
C20A—H20A	0.9700	C24B—C25B	1.540 (5)
C20A—H20B	0.9700	C24B—H24E	0.9700
C20A—H20C	0.9700	C24B—H24H	0.9700
C20A—H20D	0.9698	C25B—C26B	1.547 (5)
C21A—C22A	1.548 (6)	C25B—H25E	0.9700
C23A—C24A	1.532 (5)	C25B—H25F	0.9700
C23A—C24C	1.531 (5)	C24D—C25D	1.543 (5)
C23A—H23A	0.9700	C24D—H24F	0.9700
C23A—H23B	0.9700	C24D—H24G	0.9700
C23A—H23C	0.9701	C25D—C26B	1.525 (5)
C23A—H23D	0.9700	C25D—H25G	0.9700
C24A—C25A	1.528 (5)	C25D—H25H	0.9700
C24A—H24A	0.9700	C26B—H26E	0.9700
C24A—H24B	0.9700	C26B—H26F	0.9700
C25A—C26A	1.507 (5)	C26B—H26K	0.9700
C25A—H25A	0.9700	C26B—H26L	0.9700
C25A—H25B	0.9700	C27B—C28D	1.520 (5)
C24C—C25C	1.528 (5)	C27B—C28B	1.550 (5)
C24C—H24C	0.9700	C27B—H27C	0.9700
C24C—H24D	0.9700	C27B—H27D	0.9700
C25C—C26A	1.533 (5)	C27B—H27E	0.9701
C25C—H25C	0.9700	C27B—H27F	0.9700
C25C—H25D	0.9700	C28B—C29B	1.545 (5)
C26A—H26A	0.9700	C28B—H28C	0.9700
C26A—H26B	0.9700	C28B—H28D	0.9700
C26A—H26C	0.9700	C29B—C30B	1.543 (5)
C26A—H26D	0.9700	C29B—H29C	0.9700
C27A—C28A	1.531 (4)	C29B—H29D	0.9700
C27A—H27A	0.9700	C28D—C29D	1.538 (5)
C27A—H27B	0.9700	C28D—H28E	0.9700
C28A—C29A	1.521 (4)	C28D—H28F	0.9700
C28A—H28A	0.9700	C29D—C30B	1.540 (5)
C28A—H28B	0.9700	C29D—H29E	0.9700
C29A—C30A	1.525 (4)	C29D—H29F	0.9700
C29A—H29A	0.9700	C30B—H30C	0.9700
C29A—H29B	0.9700	C30B—H30D	0.9700
C30A—H30A	0.9700	C30B—H30E	0.9698
C30A—H30B	0.9700	C30B—H30F	0.9700
			
O2A···Cl4A^i^	2.876 (3)	O2B···Cl4B^i^	3.022 (3)
			
O3A—Er1A—O5A	88.49 (9)	O3B—Er1B—O5B	87.32 (9)
O3A—Er1A—O1A	88.56 (9)	O3B—Er1B—O1B	88.74 (9)
O5A—Er1A—O1A	87.75 (9)	O5B—Er1B—O1B	88.51 (9)
O3A—Er1A—Cl12	174.81 (7)	O3B—Er1B—Cl21	88.78 (7)
O5A—Er1A—Cl12	86.63 (7)	O5B—Er1B—Cl21	173.35 (7)
O1A—Er1A—Cl12	89.46 (7)	O1B—Er1B—Cl21	86.02 (6)
O3A—Er1A—Cl13	85.83 (6)	O3B—Er1B—Cl23	87.43 (6)
O5A—Er1A—Cl13	89.65 (7)	O5B—Er1B—Cl23	89.18 (7)
O1A—Er1A—Cl13	173.88 (7)	O1B—Er1B—Cl23	175.61 (6)
Cl12—Er1A—Cl13	95.92 (4)	Cl21—Er1B—Cl23	96.03 (3)
O3A—Er1A—Cl11	88.81 (7)	O3B—Er1B—Cl22	172.28 (7)
O5A—Er1A—Cl11	174.11 (7)	O5B—Er1B—Cl22	85.81 (7)
O1A—Er1A—Cl11	86.95 (6)	O1B—Er1B—Cl22	87.57 (7)
Cl12—Er1A—Cl11	95.88 (4)	Cl21—Er1B—Cl22	97.72 (4)
Cl13—Er1A—Cl11	95.38 (4)	Cl23—Er1B—Cl22	95.98 (4)
O1A—P1A—N2A	109.50 (15)	O1B—P1B—N2B	108.75 (15)
O1A—P1A—N3A	119.27 (16)	O1B—P1B—N3B	120.37 (16)
N2A—P1A—N3A	106.81 (17)	N2B—P1B—N3B	106.86 (17)
O1A—P1A—N1A	103.34 (14)	O1B—P1B—N1B	103.07 (14)
N2A—P1A—N1A	114.81 (17)	N2B—P1B—N1B	114.02 (16)
N3A—P1A—N1A	103.31 (15)	N3B—P1B—N1B	103.95 (16)
O3A—P2A—N5A	119.72 (17)	O3B—P2B—N5B	119.56 (15)
O3A—P2A—N6A	108.37 (15)	O3B—P2B—N6B	108.51 (15)
N5A—P2A—N6A	106.72 (19)	N5B—P2B—N6B	107.00 (16)
O3A—P2A—N4A	103.76 (14)	O3B—P2B—N4B	103.28 (14)
N5A—P2A—N4A	103.69 (17)	N5B—P2B—N4B	104.35 (15)
N6A—P2A—N4A	114.92 (16)	N6B—P2B—N4B	114.40 (16)
O5A—P3A—N8A	119.35 (19)	O5B—P3B—N9B	108.8 (2)
O5A—P3A—N9A	108.85 (17)	O5B—P3B—N8B	119.6 (2)
N8A—P3A—N9A	106.32 (19)	N9B—P3B—N8B	107.6 (3)
O5A—P3A—N7A	103.33 (14)	O5B—P3B—N7B	103.41 (15)
N8A—P3A—N7A	104.69 (18)	N9B—P3B—N7B	113.9 (2)
N9A—P3A—N7A	114.61 (18)	N8B—P3B—N7B	103.7 (2)
P1A—O1A—Er1A	151.05 (15)	P1B—O1B—Er1B	150.80 (15)
C1A—O2A—Cl4A^i^	125.4 (3)	C1B—O2B—Cl4B^i^	122.5 (3)
P2A—O3A—Er1A	153.13 (15)	P2B—O3B—Er1B	151.27 (15)
P3A—O5A—Er1A	152.74 (16)	P3B—O5B—Er1B	154.87 (16)
C1A—N1A—P1A	126.4 (3)	C1B—N1B—P1B	126.1 (3)
C1A—N1A—H1AA	116.8	C1B—N1B—H1BA	117.0
P1A—N1A—H1AA	116.8	P1B—N1B—H1BA	117.0
C3A—N2A—C6A	111.3 (3)	C3B—N2B—C6B	111.9 (3)
C3A—N2A—P1A	122.6 (2)	C3B—N2B—P1B	120.7 (2)
C6A—N2A—P1A	125.7 (3)	C6B—N2B—P1B	125.8 (3)
C10A—N3A—C7A	110.0 (3)	C10B—N3B—C7B	109.8 (3)
C10A—N3A—P1A	122.8 (3)	C10B—N3B—P1B	121.6 (3)
C7A—N3A—P1A	120.3 (3)	C7B—N3B—P1B	121.4 (3)
C11A—N4A—P2A	124.6 (3)	C11B—N4B—P2B	125.9 (3)
C11A—N4A—H4AC	117.7	C11B—N4B—H4BC	117.1
P2A—N4A—H4AC	117.7	P2B—N4B—H4BC	117.1
C16A—N5A—C13A	110.8 (3)	C13B—N5B—C16B	110.3 (3)
C16A—N5A—P2A	121.3 (3)	C13B—N5B—P2B	122.3 (2)
C13A—N5A—P2A	122.5 (3)	C16B—N5B—P2B	122.9 (3)
C20A—N6A—C17A	111.5 (3)	C17B—N6B—C20B	109.8 (3)
C20A—N6A—P2A	119.9 (3)	C17B—N6B—P2B	127.1 (3)
C17A—N6A—P2A	126.5 (3)	C20B—N6B—P2B	120.3 (3)
C21A—N7A—P3A	127.2 (3)	C21B—N7B—P3B	125.5 (3)
C21A—N7A—H7AC	116.4	C21B—N7B—H7BC	117.2
P3A—N7A—H7AC	116.4	P3B—N7B—H7BC	117.2
C26A—N8A—C23A	109.4 (3)	C23B—N8B—C26B	109.0 (4)
C26A—N8A—P3A	120.0 (3)	C23B—N8B—P3B	123.1 (4)
C23A—N8A—P3A	122.9 (3)	C26B—N8B—P3B	121.1 (4)
C30A—N9A—C27A	111.6 (3)	C30B—N9B—C27B	109.4 (4)
C30A—N9A—P3A	121.3 (3)	C30B—N9B—P3B	120.9 (3)
C27A—N9A—P3A	126.3 (3)	C27B—N9B—P3B	129.0 (4)
O2A—C1A—N1A	125.9 (3)	O2B—C1B—N1B	125.8 (4)
O2A—C1A—C2A	119.7 (3)	O2B—C1B—C2B	118.8 (3)
N1A—C1A—C2A	114.4 (3)	N1B—C1B—C2B	115.4 (3)
C1A—C2A—Cl2A	111.0 (3)	C1B—C2B—Cl1B	107.6 (3)
C1A—C2A—Cl3A	110.7 (3)	C1B—C2B—Cl2B	110.5 (3)
Cl2A—C2A—Cl3A	108.7 (2)	Cl1B—C2B—Cl2B	110.1 (2)
C1A—C2A—Cl1A	108.8 (3)	C1B—C2B—Cl3B	110.1 (3)
Cl2A—C2A—Cl1A	108.8 (2)	Cl1B—C2B—Cl3B	109.7 (2)
Cl3A—C2A—Cl1A	108.7 (2)	Cl2B—C2B—Cl3B	108.8 (2)
N2A—C3A—C4C	105.3 (6)	N2B—C3B—C4B	105.0 (3)
N2A—C3A—C4A	106.5 (8)	N2B—C3B—H3BA	110.8
C4C—C3A—C4A	17.5 (11)	C4B—C3B—H3BA	110.8
N2A—C3A—H3AA	110.4	N2B—C3B—H3BB	110.8
C4C—C3A—H3AA	125.5	C4B—C3B—H3BB	110.8
C4A—C3A—H3AA	110.4	H3BA—C3B—H3BB	108.8
N2A—C3A—H3AB	110.4	C5B—C4B—C3B	104.0 (4)
C4C—C3A—H3AB	95.3	C5B—C4B—H4BA	111.0
C4A—C3A—H3AB	110.4	C3B—C4B—H4BA	111.0
H3AA—C3A—H3AB	108.6	C5B—C4B—H4BB	111.0
N2A—C3A—H3CA	108.8	C3B—C4B—H4BB	111.0
C4C—C3A—H3CA	106.0	H4BA—C4B—H4BB	109.0
C4A—C3A—H3CA	89.4	C6B—C5B—C4B	106.2 (4)
H3AA—C3A—H3CA	23.4	C6B—C5B—H5BA	110.5
H3AB—C3A—H3CA	127.9	C4B—C5B—H5BA	110.5
N2A—C3A—H3CB	112.4	C6B—C5B—H5BB	110.5
C4C—C3A—H3CB	115.1	C4B—C5B—H5BB	110.5
C4A—C3A—H3CB	127.6	H5BA—C5B—H5BB	108.7
H3AA—C3A—H3CB	87.4	N2B—C6B—C5B	102.1 (3)
H3AB—C3A—H3CB	22.7	N2B—C6B—H6BA	111.3
H3CA—C3A—H3CB	108.9	C5B—C6B—H6BA	111.3
C3A—C4A—C5A	101.3 (11)	N2B—C6B—H6BB	111.3
C3A—C4A—H4AA	111.5	C5B—C6B—H6BB	111.3
C5A—C4A—H4AA	111.5	H6BA—C6B—H6BB	109.2
C3A—C4A—H4AB	111.5	N3B—C7B—C8D	103.6 (7)
C5A—C4A—H4AB	111.5	N3B—C7B—C8B	103.6 (8)
H4AA—C4A—H4AB	109.3	C8D—C7B—C8B	25.9 (6)
C6A—C5A—C4A	106.7 (12)	N3B—C7B—H7BA	111.0
C6A—C5A—H5AA	110.4	C8D—C7B—H7BA	131.9
C4A—C5A—H5AA	110.4	C8B—C7B—H7BA	111.0
C6A—C5A—H5AB	110.4	N3B—C7B—H7BB	111.0
C4A—C5A—H5AB	110.4	C8D—C7B—H7BB	87.5
H5AA—C5A—H5AB	108.6	C8B—C7B—H7BB	111.0
C3A—C4C—C5C	102.3 (9)	H7BA—C7B—H7BB	109.0
C3A—C4C—H4CA	111.3	N3B—C7B—H7DA	109.7
C5C—C4C—H4CA	111.3	C8D—C7B—H7DA	108.3
C3A—C4C—H4CB	111.3	C8B—C7B—H7DA	84.3
C5C—C4C—H4CB	111.3	H7BA—C7B—H7DA	28.6
H4CA—C4C—H4CB	109.2	H7BB—C7B—H7DA	130.9
C6A—C5C—C4C	103.3 (9)	N3B—C7B—H7DB	111.8
C6A—C5C—H5CA	111.1	C8D—C7B—H7DB	114.4
C4C—C5C—H5CA	111.1	C8B—C7B—H7DB	134.2
C6A—C5C—H5CB	111.1	H7BA—C7B—H7DB	82.7
C4C—C5C—H5CB	111.1	H7BB—C7B—H7DB	28.8
H5CA—C5C—H5CB	109.1	H7DA—C7B—H7DB	108.9
N2A—C6A—C5A	103.4 (8)	C7B—C8B—C9B	106.5 (11)
N2A—C6A—C5C	102.1 (6)	C7B—C8B—H8BA	110.4
C5A—C6A—C5C	26.3 (6)	C9B—C8B—H8BA	110.4
N2A—C6A—H6AA	111.1	C7B—C8B—H8BB	110.4
C5A—C6A—H6AA	111.1	C9B—C8B—H8BB	110.4
C5C—C6A—H6AA	133.0	H8BA—C8B—H8BB	108.6
N2A—C6A—H6AB	111.1	C8B—C9B—C10B	99.5 (10)
C5A—C6A—H6AB	111.1	C8B—C9B—H9BA	111.9
C5C—C6A—H6AB	87.8	C10B—C9B—H9BA	111.9
H6AA—C6A—H6AB	109.0	C8B—C9B—H9BB	111.9
N2A—C6A—H6CA	113.4	C10B—C9B—H9BB	111.9
C5A—C6A—H6CA	87.0	H9BA—C9B—H9BB	109.6
C5C—C6A—H6CA	111.0	C7B—C8D—C9D	101.3 (9)
H6AA—C6A—H6CA	24.7	C7B—C8D—H8DA	111.5
H6AB—C6A—H6CA	125.8	C9D—C8D—H8DA	111.5
N2A—C6A—H6CB	110.5	C7B—C8D—H8DB	111.5
C5A—C6A—H6CB	131.2	C9D—C8D—H8DB	111.5
C5C—C6A—H6CB	110.4	H8DA—C8D—H8DB	109.3
H6AA—C6A—H6CB	88.4	C8D—C9D—C10B	101.3 (9)
H6AB—C6A—H6CB	23.5	C8D—C9D—H9DA	111.5
H6CA—C6A—H6CB	109.2	C10B—C9D—H9DA	111.5
N3A—C7A—C8A	101.7 (6)	C8D—C9D—H9DB	111.5
N3A—C7A—C8C	103.0 (9)	C10B—C9D—H9DB	111.5
C8A—C7A—C8C	23.7 (11)	H9DA—C9D—H9DB	109.3
N3A—C7A—H7AA	111.4	N3B—C10B—C9B	107.5 (8)
C8A—C7A—H7AA	111.4	N3B—C10B—C9D	104.9 (6)
C8C—C7A—H7AA	89.5	C9B—C10B—C9D	22.7 (6)
N3A—C7A—H7AB	111.4	N3B—C10B—H10E	110.2
C8A—C7A—H7AB	111.4	C9B—C10B—H10E	110.2
C8C—C7A—H7AB	130.0	C9D—C10B—H10E	129.9
H7AA—C7A—H7AB	109.3	N3B—C10B—H10F	110.2
N3A—C7A—H7CA	117.9	C9B—C10B—H10F	110.2
C8A—C7A—H7CA	133.1	C9D—C10B—H10F	90.9
C8C—C7A—H7CA	116.5	H10E—C10B—H10F	108.5
H7AA—C7A—H7CA	32.5	N3B—C10B—H10K	111.2
H7AB—C7A—H7CA	77.8	C9B—C10B—H10K	91.1
N3A—C7A—H7CB	100.5	C9D—C10B—H10K	112.4
C8A—C7A—H7CB	85.7	H10E—C10B—H10K	20.5
C8C—C7A—H7CB	108.5	H10F—C10B—H10K	124.1
H7AA—C7A—H7CB	138.7	N3B—C10B—H10L	110.9
H7AB—C7A—H7CB	31.7	C9B—C10B—H10L	125.3
H7CA—C7A—H7CB	109.0	C9D—C10B—H10L	108.3
C9A—C8A—C7A	97.9 (8)	H10E—C10B—H10L	91.4
C9A—C8A—H8AA	112.2	H10F—C10B—H10L	18.8
C7A—C8A—H8AA	112.2	H10K—C10B—H10L	109.1
C9A—C8A—H8AB	112.2	O4B—C11B—N4B	123.6 (4)
C7A—C8A—H8AB	112.2	O4B—C11B—C12B	120.7 (4)
H8AA—C8A—H8AB	109.8	N4B—C11B—C12B	115.7 (4)
C10A—C9A—C8A	101.6 (9)	C11B—C12B—Cl6B	112.2 (3)
C10A—C9A—H9AA	111.5	C11B—C12B—Cl4B	108.7 (3)
C8A—C9A—H9AA	111.5	Cl6B—C12B—Cl4B	111.0 (3)
C10A—C9A—H9AB	111.5	C11B—C12B—Cl5B	107.4 (3)
C8A—C9A—H9AB	111.5	Cl6B—C12B—Cl5B	108.2 (3)
H9AA—C9A—H9AB	109.3	Cl4B—C12B—Cl5B	109.2 (3)
C9C—C8C—C7A	110.5 (13)	N5B—C13B—C14D	104.9 (7)
C9C—C8C—H8CA	109.5	N5B—C13B—C14B	102.1 (5)
C7A—C8C—H8CA	109.5	C14D—C13B—C14B	27.0 (6)
C9C—C8C—H8CB	109.5	N5B—C13B—H13E	111.3
C7A—C8C—H8CB	109.5	C14D—C13B—H13E	85.8
H8CA—C8C—H8CB	108.1	C14B—C13B—H13E	111.3
C10A—C9C—C8C	99.7 (12)	N5B—C13B—H13F	111.3
C10A—C9C—H9CA	111.8	C14D—C13B—H13F	131.1
C8C—C9C—H9CA	111.8	C14B—C13B—H13F	111.3
C10A—C9C—H9CB	111.8	H13E—C13B—H13F	109.2
C8C—C9C—H9CB	111.8	N5B—C13B—H13K	110.0
H9CA—C9C—H9CB	109.6	C14D—C13B—H13K	108.0
N3A—C10A—C9C	109.8 (8)	C14B—C13B—H13K	131.9
N3A—C10A—C9A	102.4 (6)	H13E—C13B—H13K	23.1
C9C—C10A—C9A	24.4 (6)	H13F—C13B—H13K	89.4
N3A—C10A—H10A	111.3	N5B—C13B—H13L	111.0
C9C—C10A—H10A	87.1	C14D—C13B—H13L	114.3
C9A—C10A—H10A	111.3	C14B—C13B—H13L	91.4
N3A—C10A—H10B	111.3	H13E—C13B—H13L	125.4
C9C—C10A—H10B	125.3	H13F—C13B—H13L	21.1
C9A—C10A—H10B	111.3	H13K—C13B—H13L	108.5
H10A—C10A—H10B	109.2	C15B—C14B—C13B	98.0 (6)
N3A—C10A—H10C	102.4	C15B—C14B—H14E	112.2
C9C—C10A—H10C	105.7	C13B—C14B—H14E	112.2
C9A—C10A—H10C	129.9	C15B—C14B—H14F	112.2
H10A—C10A—H10C	18.6	C13B—C14B—H14F	112.2
H10B—C10A—H10C	99.0	H14E—C14B—H14F	109.8
N3A—C10A—H10D	116.4	C14B—C15B—C16B	100.5 (7)
C9C—C10A—H10D	113.0	C14B—C15B—H15E	111.7
C9A—C10A—H10D	98.2	C16B—C15B—H15E	111.7
H10A—C10A—H10D	115.5	C14B—C15B—H15F	111.7
H10B—C10A—H10D	13.1	C16B—C15B—H15F	111.7
H10C—C10A—H10D	108.4	H15E—C15B—H15F	109.4
O4A—C11A—N4A	125.6 (4)	C15D—C14D—C13B	106.7 (10)
O4A—C11A—C12A	118.6 (4)	C15D—C14D—H14G	110.4
N4A—C11A—C12A	115.8 (4)	C13B—C14D—H14G	110.4
C11A—C12A—Cl4A	110.2 (3)	C15D—C14D—H14H	110.4
C11A—C12A—Cl6A	112.4 (3)	C13B—C14D—H14H	110.4
Cl4A—C12A—Cl6A	110.3 (3)	H14G—C14D—H14H	108.6
C11A—C12A—Cl5A	106.0 (3)	C14D—C15D—C16B	101.8 (9)
Cl4A—C12A—Cl5A	111.3 (3)	C14D—C15D—H15G	111.4
Cl6A—C12A—Cl5A	106.5 (3)	C16B—C15D—H15G	111.4
N5A—C13A—C14C	102.5 (4)	C14D—C15D—H15H	111.4
N5A—C13A—C14A	102.2 (4)	C16B—C15D—H15H	111.4
C14C—C13A—C14A	24.7 (5)	H15G—C15D—H15H	109.3
N5A—C13A—H13A	111.3	N5B—C16B—C15D	107.1 (7)
C14C—C13A—H13A	88.8	N5B—C16B—C15B	101.5 (5)
C14A—C13A—H13A	111.3	C15D—C16B—C15B	23.9 (9)
N5A—C13A—H13B	111.3	N5B—C16B—H16E	111.5
C14C—C13A—H13B	131.2	C15D—C16B—H16E	88.1
C14A—C13A—H13B	111.3	C15B—C16B—H16E	111.5
H13A—C13A—H13B	109.2	N5B—C16B—H16F	111.5
N5A—C13A—H13C	116.4	C15D—C16B—H16F	126.9
C14C—C13A—H13C	114.9	C15B—C16B—H16F	111.5
C14A—C13A—H13C	132.5	H16E—C16B—H16F	109.3
H13A—C13A—H13C	30.2	N5B—C16B—H16K	109.8
H13B—C13A—H13C	80.3	C15D—C16B—H16K	105.9
N5A—C13A—H13D	103.1	C15B—C16B—H16K	128.6
C14C—C13A—H13D	109.4	H16E—C16B—H16K	18.6
C14A—C13A—H13D	85.7	H16F—C16B—H16K	93.8
H13A—C13A—H13D	136.4	N5B—C16B—H16L	110.4
H13B—C13A—H13D	30.3	C15D—C16B—H16L	115.0
H13C—C13A—H13D	109.7	C15B—C16B—H16L	96.9
C13A—C14A—C15A	95.4 (5)	H16E—C16B—H16L	122.3
C13A—C14A—H14A	112.7	H16F—C16B—H16L	15.8
C15A—C14A—H14A	112.7	H16K—C16B—H16L	108.4
C13A—C14A—H14B	112.7	N6B—C17B—C18B	103.9 (8)
C15A—C14A—H14B	112.7	N6B—C17B—C18D	101.5 (5)
H14A—C14A—H14B	110.2	C18B—C17B—C18D	20.2 (11)
C14A—C15A—C16A	102.3 (6)	N6B—C17B—H17E	111.0
C14A—C15A—H15A	111.3	C18B—C17B—H17E	111.0
C16A—C15A—H15A	111.3	C18D—C17B—H17E	93.9
C14A—C15A—H15B	111.3	N6B—C17B—H17F	111.0
C16A—C15A—H15B	111.3	C18B—C17B—H17F	111.0
H15A—C15A—H15B	109.2	C18D—C17B—H17F	129.0
C13A—C14C—C15C	112.9 (6)	H17E—C17B—H17F	109.0
C13A—C14C—H14C	109.0	N6B—C17B—H17K	112.1
C15C—C14C—H14C	109.0	C18B—C17B—H17K	126.9
C13A—C14C—H14D	109.0	C18D—C17B—H17K	111.9
C15C—C14C—H14D	109.0	H17E—C17B—H17K	19.3
H14C—C14C—H14D	107.8	H17F—C17B—H17K	91.4
C16A—C15C—C14C	100.2 (5)	N6B—C17B—H17L	112.8
C16A—C15C—H15C	111.7	C18B—C17B—H17L	89.4
C14C—C15C—H15C	111.7	C18D—C17B—H17L	108.5
C16A—C15C—H15D	111.7	H17E—C17B—H17L	124.7
C14C—C15C—H15D	111.7	H17F—C17B—H17L	22.3
H15C—C15C—H15D	109.5	H17K—C17B—H17L	109.9
N5A—C16A—C15C	109.6 (4)	C19B—C18B—C17B	113.5 (12)
N5A—C16A—C15A	98.7 (4)	C19B—C18B—H18E	108.9
C15C—C16A—C15A	23.5 (4)	C17B—C18B—H18E	108.9
N5A—C16A—H16A	112.0	C19B—C18B—H18F	108.9
C15C—C16A—H16A	88.6	C17B—C18B—H18F	108.9
C15A—C16A—H16A	112.0	H18E—C18B—H18F	107.7
N5A—C16A—H16B	112.0	C20B—C19B—C18B	100.8 (11)
C15C—C16A—H16B	122.7	C20B—C19B—H19E	111.6
C15A—C16A—H16B	112.0	C18B—C19B—H19E	111.6
H16A—C16A—H16B	109.7	C20B—C19B—H19F	111.6
N5A—C16A—H16C	102.0	C18B—C19B—H19F	111.6
C15C—C16A—H16C	106.0	H19E—C19B—H19F	109.4
C15A—C16A—H16C	129.4	C17B—C18D—C19D	95.0 (8)
H16A—C16A—H16C	17.8	C17B—C18D—H18G	112.7
H16B—C16A—H16C	102.0	C19D—C18D—H18G	112.7
N5A—C16A—H16D	116.3	C17B—C18D—H18H	112.7
C15C—C16A—H16D	113.6	C19D—C18D—H18H	112.7
C15A—C16A—H16D	102.8	H18G—C18D—H18H	110.2
H16A—C16A—H16D	113.6	C20B—C19D—C18D	101.1 (8)
H16B—C16A—H16D	9.4	C20B—C19D—H19G	111.5
H16C—C16A—H16D	108.3	C18D—C19D—H19G	111.6
N6A—C17A—C18C	101.4 (8)	C20B—C19D—H19H	111.5
N6A—C17A—C18A	99.5 (6)	C18D—C19D—H19H	111.5
C18C—C17A—C18A	36.2 (6)	H19G—C19D—H19H	109.4
N6A—C17A—H17A	111.9	N6B—C20B—C19B	111.5 (8)
C18C—C17A—H17A	138.7	N6B—C20B—C19D	99.1 (6)
C18A—C17A—H17A	111.9	C19B—C20B—C19D	22.0 (9)
N6A—C17A—H17B	111.9	N6B—C20B—H20E	109.3
C18C—C17A—H17B	78.0	C19B—C20B—H20E	109.3
C18A—C17A—H17B	111.9	C19D—C20B—H20E	98.2
H17A—C17A—H17B	109.6	N6B—C20B—H20F	109.3
N6A—C17A—H17C	114.4	C19B—C20B—H20F	109.3
C18C—C17A—H17C	118.5	C19D—C20B—H20F	131.3
C18A—C17A—H17C	88.0	H20E—C20B—H20F	108.0
H17A—C17A—H17C	24.2	N6B—C20B—H20K	110.7
H17B—C17A—H17C	125.1	C19B—C20B—H20K	120.0
N6A—C17A—H17D	106.4	C19D—C20B—H20K	111.8
C18C—C17A—H17D	106.4	H20E—C20B—H20K	14.5
C18A—C17A—H17D	139.0	H20F—C20B—H20K	94.3
H17A—C17A—H17D	87.3	N6B—C20B—H20L	112.3
H17B—C17A—H17D	28.4	C19B—C20B—H20L	91.6
H17C—C17A—H17D	108.6	C19D—C20B—H20L	113.4
C17A—C18A—C19A	99.4 (10)	H20E—C20B—H20L	121.5
C17A—C18A—H18A	111.9	H20F—C20B—H20L	18.8
C19A—C18A—H18A	111.9	H20K—C20B—H20L	109.3
C17A—C18A—H18B	111.9	O6B—C21B—N7B	126.3 (5)
C19A—C18A—H18B	111.9	O6B—C21B—C22B	121.4 (4)
H18A—C18A—H18B	109.6	N7B—C21B—C22B	112.3 (4)
C20A—C19A—C18A	99.0 (9)	C21B—C22B—Cl8B	111.9 (3)
C20A—C19A—H19A	112.0	C21B—C22B—Cl9B	112.5 (3)
C18A—C19A—H19A	112.0	Cl8B—C22B—Cl9B	107.8 (3)
C20A—C19A—H19B	112.0	C21B—C22B—Cl7B	111.1 (4)
C18A—C19A—H19B	112.0	Cl8B—C22B—Cl7B	106.8 (3)
H19A—C19A—H19B	109.6	Cl9B—C22B—Cl7B	106.4 (3)
C17A—C18C—C19C	108.6 (11)	N8B—C23B—C24B	113.6 (6)
C17A—C18C—H18C	110.0	N8B—C23B—C24D	94.4 (6)
C19C—C18C—H18C	110.0	C24B—C23B—C24D	23.2 (6)
C17A—C18C—H18D	110.0	N8B—C23B—H23E	108.9
C19C—C18C—H18D	110.0	C24B—C23B—H23E	108.9
H18C—C18C—H18D	108.4	C24D—C23B—H23E	129.9
C20A—C19C—C18C	101.0 (12)	N8B—C23B—H23F	108.9
C20A—C19C—H19C	111.6	C24B—C23B—H23F	108.8
C18C—C19C—H19C	111.6	C24D—C23B—H23F	105.4
C20A—C19C—H19D	111.6	H23E—C23B—H23F	107.7
C18C—C19C—H19D	111.6	N8B—C23B—H23K	111.3
H19C—C19C—H19D	109.4	C24B—C23B—H23K	95.3
N6A—C20A—C19C	108.0 (7)	C24D—C23B—H23K	115.5
N6A—C20A—C19A	101.1 (7)	H23E—C23B—H23K	14.6
C19C—C20A—C19A	16.0 (14)	H23F—C23B—H23K	118.6
N6A—C20A—H20A	111.5	N8B—C23B—H23L	109.5
C19C—C20A—H20A	119.8	C24B—C23B—H23L	117.8
C19A—C20A—H20A	111.5	C24D—C23B—H23L	116.8
N6A—C20A—H20B	111.5	H23E—C23B—H23L	96.7
C19C—C20A—H20B	95.6	H23F—C23B—H23L	11.8
C19A—C20A—H20B	111.5	H23K—C23B—H23L	108.5
H20A—C20A—H20B	109.4	C23B—C24B—C25B	100.7 (7)
N6A—C20A—H20C	105.8	C23B—C24B—H24E	111.6
C19C—C20A—H20C	106.4	C25B—C24B—H24E	111.6
C19A—C20A—H20C	95.1	C23B—C24B—H24H	111.7
H20A—C20A—H20C	19.8	C25B—C24B—H24H	111.6
H20B—C20A—H20C	127.7	H24E—C24B—H24H	109.4
N6A—C20A—H20D	114.8	C24B—C25B—C26B	112.4 (8)
C19C—C20A—H20D	112.7	C24B—C25B—H25E	109.1
C19A—C20A—H20D	128.0	C26B—C25B—H25E	109.1
H20A—C20A—H20D	89.4	C24B—C25B—H25F	109.1
H20B—C20A—H20D	21.2	C26B—C25B—H25F	109.1
H20C—C20A—H20D	108.7	H25E—C25B—H25F	107.9
O6A—C21A—N7A	125.4 (4)	C25D—C24D—C23B	104.5 (8)
O6A—C21A—C22A	120.5 (4)	C25D—C24D—H24F	110.8
N7A—C21A—C22A	114.2 (4)	C23B—C24D—H24F	110.8
C21A—C22A—Cl9A	111.2 (3)	C25D—C24D—H24G	110.9
C21A—C22A—Cl8A	110.8 (3)	C23B—C24D—H24G	110.9
Cl9A—C22A—Cl8A	109.9 (3)	H24F—C24D—H24G	108.9
C21A—C22A—Cl7A	107.3 (3)	C26B—C25D—C24D	86.9 (7)
Cl9A—C22A—Cl7A	109.5 (3)	C26B—C25D—H25G	114.2
Cl8A—C22A—Cl7A	108.0 (3)	C24D—C25D—H25G	114.2
N8A—C23A—C24A	105.6 (3)	C26B—C25D—H25H	114.2
N8A—C23A—C24C	106.4 (5)	C24D—C25D—H25H	114.1
C24A—C23A—C24C	26.7 (5)	H25G—C25D—H25H	111.3
N8A—C23A—H23A	110.6	N8B—C26B—C25D	105.1 (6)
C24A—C23A—H23A	110.6	N8B—C26B—C25B	104.3 (6)
C24C—C23A—H23A	131.1	C25D—C26B—C25B	23.7 (6)
N8A—C23A—H23B	110.6	N8B—C26B—H26E	110.9
C24A—C23A—H23B	110.6	C25D—C26B—H26E	129.5
C24C—C23A—H23B	86.0	C25B—C26B—H26E	110.9
H23A—C23A—H23B	108.8	N8B—C26B—H26F	110.9
N8A—C23A—H23C	102.0	C25D—C26B—H26F	89.1
C24A—C23A—H23C	106.7	C25B—C26B—H26F	110.9
C24C—C23A—H23C	130.6	H26E—C26B—H26F	108.9
H23A—C23A—H23C	11.8	N8B—C26B—H26K	112.5
H23B—C23A—H23C	120.2	C25D—C26B—H26K	117.1
N8A—C23A—H23D	115.8	C25B—C26B—H26K	97.0
C24A—C23A—H23D	116.8	H26E—C26B—H26K	14.4
C24C—C23A—H23D	94.2	H26F—C26B—H26K	119.4
H23A—C23A—H23D	97.3	N8B—C26B—H26L	111.3
H23B—C23A—H23D	11.5	C25D—C26B—H26L	101.2
H23C—C23A—H23D	108.7	C25B—C26B—H26L	121.9
C25A—C24A—C23A	104.3 (4)	H26E—C26B—H26L	97.5
C25A—C24A—H24A	110.9	H26F—C26B—H26L	12.8
C23A—C24A—H24A	110.9	H26K—C26B—H26L	109.1
C25A—C24A—H24B	110.9	N9B—C27B—C28D	102.4 (5)
C23A—C24A—H24B	110.9	N9B—C27B—C28B	98.7 (5)
H24A—C24A—H24B	108.9	C28D—C27B—C28B	26.0 (5)
C24A—C25A—C26A	104.9 (4)	N9B—C27B—H27C	112.0
C24A—C25A—H25A	110.8	C28D—C27B—H27C	130.9
C26A—C25A—H25A	110.8	C28B—C27B—H27C	112.0
C24A—C25A—H25B	110.8	N9B—C27B—H27D	112.0
C26A—C25A—H25B	110.8	C28D—C27B—H27D	87.2
H25A—C25A—H25B	108.8	C28B—C27B—H27D	112.0
C25C—C24C—C23A	100.1 (6)	H27C—C27B—H27D	109.7
C25C—C24C—H24C	111.8	N9B—C27B—H27E	109.7
C23A—C24C—H24C	111.8	C28D—C27B—H27E	112.6
C25C—C24C—H24D	111.8	C28B—C27B—H27E	90.4
C23A—C24C—H24D	111.8	H27C—C27B—H27E	23.0
H24C—C24C—H24D	109.5	H27D—C27B—H27E	128.0
C26A—C25C—C24C	106.2 (6)	N9B—C27B—H27F	110.8
C26A—C25C—H25C	110.5	C28D—C27B—H27F	112.6
C24C—C25C—H25C	110.5	C28B—C27B—H27F	135.7
C26A—C25C—H25D	110.5	H27C—C27B—H27F	87.1
C24C—C25C—H25D	110.5	H27D—C27B—H27F	26.3
H25C—C25C—H25D	108.7	H27E—C27B—H27F	108.7
N8A—C26A—C25C	103.5 (5)	C29B—C28B—C27B	95.9 (6)
N8A—C26A—C25A	102.2 (4)	C29B—C28B—H28C	112.6
C25C—C26A—C25A	22.3 (6)	C27B—C28B—H28C	112.6
N8A—C26A—H26A	111.3	C29B—C28B—H28D	112.6
C25C—C26A—H26A	90.7	C27B—C28B—H28D	112.6
C25A—C26A—H26A	111.3	H28C—C28B—H28D	110.1
N8A—C26A—H26B	111.3	C30B—C29B—C28B	98.2 (6)
C25C—C26A—H26B	128.8	C30B—C29B—H29C	112.1
C25A—C26A—H26B	111.3	C28B—C29B—H29C	112.1
H26A—C26A—H26B	109.2	C30B—C29B—H29D	112.1
N8A—C26A—H26C	119.3	C28B—C29B—H29D	112.1
C25C—C26A—H26C	113.6	H29C—C29B—H29D	109.8
C25A—C26A—H26C	129.4	C27B—C28D—C29D	114.6 (6)
H26A—C26A—H26C	29.2	C27B—C28D—H28E	108.6
H26B—C26A—H26C	80.5	C29D—C28D—H28E	108.6
N8A—C26A—H26D	99.0	C27B—C28D—H28F	108.6
C25C—C26A—H26D	112.2	C29D—C28D—H28F	108.6
C25A—C26A—H26D	90.8	H28E—C28D—H28F	107.6
H26A—C26A—H26D	136.6	C28D—C29D—C30B	98.1 (6)
H26B—C26A—H26D	28.1	C28D—C29D—H29E	112.2
H26C—C26A—H26D	108.4	C30B—C29D—H29E	112.2
N9A—C27A—C28A	101.7 (4)	C28D—C29D—H29F	112.2
N9A—C27A—H27A	111.4	C30B—C29D—H29F	112.2
C28A—C27A—H27A	111.4	H29E—C29D—H29F	109.8
N9A—C27A—H27B	111.4	N9B—C30B—C29D	111.7 (5)
C28A—C27A—H27B	111.4	N9B—C30B—C29B	101.0 (5)
H27A—C27A—H27B	109.3	C29D—C30B—C29B	24.1 (6)
C29A—C28A—C27A	105.9 (5)	N9B—C30B—H30C	111.6
C29A—C28A—H28A	110.5	C29D—C30B—H30C	122.6
C27A—C28A—H28A	110.5	C29B—C30B—H30C	111.6
C29A—C28A—H28B	110.5	N9B—C30B—H30D	111.6
C27A—C28A—H28B	110.5	C29D—C30B—H30D	87.4
H28A—C28A—H28B	108.7	C29B—C30B—H30D	111.6
C28A—C29A—C30A	102.3 (5)	H30C—C30B—H30D	109.4
C28A—C29A—H29A	111.3	N9B—C30B—H30E	110.1
C30A—C29A—H29A	111.3	C29D—C30B—H30E	113.0
C28A—C29A—H29B	111.3	C29B—C30B—H30E	98.8
C30A—C29A—H29B	111.3	H30C—C30B—H30E	14.1
H29A—C29A—H29B	109.2	H30D—C30B—H30E	121.3
N9A—C30A—C29A	106.4 (4)	N9B—C30B—H30F	109.2
N9A—C30A—H30A	110.4	C29D—C30B—H30F	104.4
C29A—C30A—H30A	110.4	C29B—C30B—H30F	128.4
N9A—C30A—H30B	110.4	H30C—C30B—H30F	95.0
C29A—C30A—H30B	110.4	H30D—C30B—H30F	17.9
H30A—C30A—H30B	108.6	H30E—C30B—H30F	108.2
			
N2A—P1A—O1A—Er1A	170.1 (3)	N2B—P1B—O1B—Er1B	170.9 (3)
N3A—P1A—O1A—Er1A	−66.5 (4)	N3B—P1B—O1B—Er1B	−65.5 (4)
N1A—P1A—O1A—Er1A	47.3 (4)	N1B—P1B—O1B—Er1B	49.5 (3)
O3A—Er1A—O1A—P1A	−115.9 (3)	O3B—Er1B—O1B—P1B	−118.2 (3)
O5A—Er1A—O1A—P1A	155.5 (3)	O5B—Er1B—O1B—P1B	154.5 (3)
Cl12—Er1A—O1A—P1A	68.9 (3)	Cl21—Er1B—O1B—P1B	−29.3 (3)
Cl13—Er1A—O1A—P1A	−139.6 (5)	Cl23—Er1B—O1B—P1B	−147.4 (6)
Cl11—Er1A—O1A—P1A	−27.0 (3)	Cl22—Er1B—O1B—P1B	68.6 (3)
N5A—P2A—O3A—Er1A	−69.3 (4)	N5B—P2B—O3B—Er1B	−64.3 (3)
N6A—P2A—O3A—Er1A	168.1 (3)	N6B—P2B—O3B—Er1B	172.7 (3)
N4A—P2A—O3A—Er1A	45.6 (4)	N4B—P2B—O3B—Er1B	51.0 (3)
O5A—Er1A—O3A—P2A	−112.2 (3)	O5B—Er1B—O3B—P2B	−117.8 (3)
O1A—Er1A—O3A—P2A	160.0 (3)	O1B—Er1B—O3B—P2B	153.6 (3)
Cl12—Er1A—O3A—P2A	−132.3 (6)	Cl21—Er1B—O3B—P2B	67.6 (3)
Cl13—Er1A—O3A—P2A	−22.4 (3)	Cl23—Er1B—O3B—P2B	−28.5 (3)
Cl11—Er1A—O3A—P2A	73.0 (3)	Cl22—Er1B—O3B—P2B	−145.0 (3)
N8A—P3A—O5A—Er1A	−72.3 (4)	N9B—P3B—O5B—Er1B	168.0 (4)
N9A—P3A—O5A—Er1A	165.5 (3)	N8B—P3B—O5B—Er1B	−67.9 (5)
N7A—P3A—O5A—Er1A	43.3 (4)	N7B—P3B—O5B—Er1B	46.6 (4)
O3A—Er1A—O5A—P3A	155.4 (3)	O3B—Er1B—O5B—P3B	156.4 (4)
O1A—Er1A—O5A—P3A	−116.0 (3)	O1B—Er1B—O5B—P3B	−114.8 (4)
Cl12—Er1A—O5A—P3A	−26.4 (3)	Cl21—Er1B—O5B—P3B	−149.5 (4)
Cl13—Er1A—O5A—P3A	69.6 (3)	Cl23—Er1B—O5B—P3B	68.9 (4)
Cl11—Er1A—O5A—P3A	−141.8 (5)	Cl22—Er1B—O5B—P3B	−27.1 (4)
O1A—P1A—N1A—C1A	171.2 (3)	O1B—P1B—N1B—C1B	169.9 (3)
N2A—P1A—N1A—C1A	52.0 (4)	N2B—P1B—N1B—C1B	52.2 (4)
N3A—P1A—N1A—C1A	−63.9 (3)	N3B—P1B—N1B—C1B	−63.8 (3)
O1A—P1A—N2A—C3A	−17.9 (4)	O1B—P1B—N2B—C3B	−32.5 (4)
N3A—P1A—N2A—C3A	−148.4 (3)	N3B—P1B—N2B—C3B	−163.9 (3)
N1A—P1A—N2A—C3A	97.8 (3)	N1B—P1B—N2B—C3B	81.8 (3)
O1A—P1A—N2A—C6A	169.7 (4)	O1B—P1B—N2B—C6B	163.3 (3)
N3A—P1A—N2A—C6A	39.3 (4)	N3B—P1B—N2B—C6B	32.0 (4)
N1A—P1A—N2A—C6A	−74.6 (4)	N1B—P1B—N2B—C6B	−82.3 (3)
O1A—P1A—N3A—C10A	75.7 (3)	O1B—P1B—N3B—C10B	75.7 (3)
N2A—P1A—N3A—C10A	−159.7 (3)	N2B—P1B—N3B—C10B	−159.7 (3)
N1A—P1A—N3A—C10A	−38.2 (3)	N1B—P1B—N3B—C10B	−38.8 (3)
O1A—P1A—N3A—C7A	−72.1 (4)	O1B—P1B—N3B—C7B	−71.5 (4)
N2A—P1A—N3A—C7A	52.6 (4)	N2B—P1B—N3B—C7B	53.0 (4)
N1A—P1A—N3A—C7A	174.1 (3)	N1B—P1B—N3B—C7B	173.9 (3)
O3A—P2A—N4A—C11A	170.8 (3)	O3B—P2B—N4B—C11B	166.8 (3)
N5A—P2A—N4A—C11A	−63.4 (4)	N5B—P2B—N4B—C11B	−67.5 (4)
N6A—P2A—N4A—C11A	52.7 (4)	N6B—P2B—N4B—C11B	49.1 (4)
O3A—P2A—N5A—C16A	78.0 (4)	O3B—P2B—N5B—C13B	−72.1 (4)
N6A—P2A—N5A—C16A	−158.6 (3)	N6B—P2B—N5B—C13B	51.6 (4)
N4A—P2A—N5A—C16A	−36.9 (4)	N4B—P2B—N5B—C13B	173.2 (3)
O3A—P2A—N5A—C13A	−73.9 (4)	O3B—P2B—N5B—C16B	81.7 (3)
N6A—P2A—N5A—C13A	49.5 (4)	N6B—P2B—N5B—C16B	−154.6 (3)
N4A—P2A—N5A—C13A	171.2 (3)	N4B—P2B—N5B—C16B	−33.0 (4)
O3A—P2A—N6A—C20A	−34.0 (4)	O3B—P2B—N6B—C17B	164.7 (3)
N5A—P2A—N6A—C20A	−164.2 (3)	N5B—P2B—N6B—C17B	34.4 (4)
N4A—P2A—N6A—C20A	81.5 (3)	N4B—P2B—N6B—C17B	−80.6 (4)
O3A—P2A—N6A—C17A	164.0 (3)	O3B—P2B—N6B—C20B	−36.4 (4)
N5A—P2A—N6A—C17A	33.8 (4)	N5B—P2B—N6B—C20B	−166.6 (3)
N4A—P2A—N6A—C17A	−80.5 (4)	N4B—P2B—N6B—C20B	78.3 (3)
O5A—P3A—N7A—C21A	167.8 (3)	O5B—P3B—N7B—C21B	172.2 (4)
N8A—P3A—N7A—C21A	−66.6 (4)	N9B—P3B—N7B—C21B	54.4 (4)
N9A—P3A—N7A—C21A	49.5 (4)	N8B—P3B—N7B—C21B	−62.3 (4)
O5A—P3A—N8A—C26A	−68.1 (4)	O5B—P3B—N8B—C23B	76.3 (5)
N9A—P3A—N8A—C26A	55.3 (4)	N9B—P3B—N8B—C23B	−159.1 (4)
N7A—P3A—N8A—C26A	177.0 (3)	N7B—P3B—N8B—C23B	−38.1 (5)
O5A—P3A—N8A—C23A	77.8 (4)	O5B—P3B—N8B—C26B	−71.6 (5)
N9A—P3A—N8A—C23A	−158.7 (4)	N9B—P3B—N8B—C26B	53.1 (5)
N7A—P3A—N8A—C23A	−37.0 (4)	N7B—P3B—N8B—C26B	174.0 (4)
O5A—P3A—N9A—C30A	−27.0 (4)	O5B—P3B—N9B—C30B	−26.4 (5)
N8A—P3A—N9A—C30A	−156.7 (4)	N8B—P3B—N9B—C30B	−157.3 (4)
N7A—P3A—N9A—C30A	88.2 (4)	N7B—P3B—N9B—C30B	88.3 (4)
O5A—P3A—N9A—C27A	164.2 (4)	O5B—P3B—N9B—C27B	164.2 (4)
N8A—P3A—N9A—C27A	34.4 (4)	N8B—P3B—N9B—C27B	33.3 (5)
N7A—P3A—N9A—C27A	−80.7 (4)	N7B—P3B—N9B—C27B	−81.1 (5)
Cl4A^i^—O2A—C1A—N1A	96.5 (4)	Cl4B^i^—O2B—C1B—N1B	90.8 (4)
Cl4A^i^—O2A—C1A—C2A	−81.7 (4)	Cl4B^i^—O2B—C1B—C2B	−88.5 (4)
P1A—N1A—C1A—O2A	−7.6 (6)	P1B—N1B—C1B—O2B	−8.9 (6)
P1A—N1A—C1A—C2A	170.6 (3)	P1B—N1B—C1B—C2B	170.4 (3)
O2A—C1A—C2A—Cl2A	−8.6 (5)	O2B—C1B—C2B—Cl1B	107.7 (4)
N1A—C1A—C2A—Cl2A	173.0 (3)	N1B—C1B—C2B—Cl1B	−71.6 (4)
O2A—C1A—C2A—Cl3A	−129.5 (4)	O2B—C1B—C2B—Cl2B	−132.1 (3)
N1A—C1A—C2A—Cl3A	52.2 (4)	N1B—C1B—C2B—Cl2B	48.6 (4)
O2A—C1A—C2A—Cl1A	111.0 (4)	O2B—C1B—C2B—Cl3B	−11.8 (5)
N1A—C1A—C2A—Cl1A	−67.3 (4)	N1B—C1B—C2B—Cl3B	168.8 (3)
C6A—N2A—C3A—C4C	3.9 (8)	C6B—N2B—C3B—C4B	−3.8 (5)
P1A—N2A—C3A—C4C	−169.4 (7)	P1B—N2B—C3B—C4B	−170.0 (3)
C6A—N2A—C3A—C4A	−14.2 (9)	N2B—C3B—C4B—C5B	22.4 (5)
P1A—N2A—C3A—C4A	172.4 (8)	C3B—C4B—C5B—C6B	−33.2 (6)
N2A—C3A—C4A—C5A	28.4 (13)	C3B—N2B—C6B—C5B	−16.2 (5)
C4C—C3A—C4A—C5A	−60 (4)	P1B—N2B—C6B—C5B	149.1 (3)
C3A—C4A—C5A—C6A	−32.8 (15)	C4B—C5B—C6B—N2B	30.1 (5)
N2A—C3A—C4C—C5C	−26.4 (11)	C10B—N3B—C7B—C8D	18.9 (7)
C4A—C3A—C4C—C5C	70 (4)	P1B—N3B—C7B—C8D	169.6 (6)
C3A—C4C—C5C—C6A	38.9 (12)	C10B—N3B—C7B—C8B	−7.8 (8)
C3A—N2A—C6A—C5A	−6.6 (7)	P1B—N3B—C7B—C8B	142.9 (7)
P1A—N2A—C6A—C5A	166.5 (6)	N3B—C7B—C8B—C9B	28.1 (13)
C3A—N2A—C6A—C5C	20.4 (7)	C8D—C7B—C8B—C9B	−65 (2)
P1A—N2A—C6A—C5C	−166.5 (6)	C7B—C8B—C9B—C10B	−35.9 (15)
C4A—C5A—C6A—N2A	24.9 (12)	N3B—C7B—C8D—C9D	−38.3 (11)
C4A—C5A—C6A—C5C	−65 (3)	C8B—C7B—C8D—C9D	55 (2)
C4C—C5C—C6A—N2A	−36.2 (10)	C7B—C8D—C9D—C10B	42.7 (12)
C4C—C5C—C6A—C5A	60 (2)	C7B—N3B—C10B—C9B	−15.2 (7)
C10A—N3A—C7A—C8A	23.1 (7)	P1B—N3B—C10B—C9B	−165.8 (6)
P1A—N3A—C7A—C8A	174.6 (6)	C7B—N3B—C10B—C9D	8.3 (7)
C10A—N3A—C7A—C8C	−1.1 (10)	P1B—N3B—C10B—C9D	−142.3 (6)
P1A—N3A—C7A—C8C	150.4 (9)	C8B—C9B—C10B—N3B	31.1 (12)
N3A—C7A—C8A—C9A	−44.3 (9)	C8B—C9B—C10B—C9D	−56 (2)
C8C—C7A—C8A—C9A	51 (3)	C8D—C9D—C10B—N3B	−31.9 (10)
C7A—C8A—C9A—C10A	50.2 (10)	C8D—C9D—C10B—C9B	68 (3)
N3A—C7A—C8C—C9C	17.6 (18)	P2B—N4B—C11B—O4B	−8.4 (6)
C8A—C7A—C8C—C9C	−72 (3)	P2B—N4B—C11B—C12B	169.0 (3)
C7A—C8C—C9C—C10A	−25.9 (19)	O4B—C11B—C12B—Cl6B	−134.5 (4)
C7A—N3A—C10A—C9C	−15.8 (8)	N4B—C11B—C12B—Cl6B	48.0 (5)
P1A—N3A—C10A—C9C	−166.4 (7)	O4B—C11B—C12B—Cl4B	−11.3 (6)
C7A—N3A—C10A—C9A	8.4 (7)	N4B—C11B—C12B—Cl4B	171.2 (3)
P1A—N3A—C10A—C9A	−142.2 (6)	O4B—C11B—C12B—Cl5B	106.8 (4)
C8C—C9C—C10A—N3A	24.9 (14)	N4B—C11B—C12B—Cl5B	−70.8 (4)
C8C—C9C—C10A—C9A	−51 (3)	C16B—N5B—C13B—C14D	−7.0 (8)
C8A—C9A—C10A—N3A	−36.8 (9)	P2B—N5B—C13B—C14D	149.7 (7)
C8A—C9A—C10A—C9C	74 (3)	C16B—N5B—C13B—C14B	20.6 (6)
P2A—N4A—C11A—O4A	−7.6 (6)	P2B—N5B—C13B—C14B	177.4 (5)
P2A—N4A—C11A—C12A	169.9 (3)	N5B—C13B—C14B—C15B	−44.0 (8)
O4A—C11A—C12A—Cl4A	−16.1 (6)	C14D—C13B—C14B—C15B	55.1 (17)
N4A—C11A—C12A—Cl4A	166.2 (3)	C13B—C14B—C15B—C16B	51.4 (9)
O4A—C11A—C12A—Cl6A	−139.6 (4)	N5B—C13B—C14D—C15D	23.8 (14)
N4A—C11A—C12A—Cl6A	42.6 (5)	C14B—C13B—C14D—C15D	−63.6 (19)
O4A—C11A—C12A—Cl5A	104.4 (4)	C13B—C14D—C15D—C16B	−30.4 (17)
N4A—C11A—C12A—Cl5A	−73.4 (4)	C13B—N5B—C16B—C15D	−12.3 (9)
C16A—N5A—C13A—C14C	−3.0 (6)	P2B—N5B—C16B—C15D	−168.9 (8)
P2A—N5A—C13A—C14C	151.4 (5)	C13B—N5B—C16B—C15B	11.7 (7)
C16A—N5A—C13A—C14A	22.3 (6)	P2B—N5B—C16B—C15B	−144.9 (6)
P2A—N5A—C13A—C14A	176.7 (4)	C14D—C15D—C16B—N5B	26.0 (15)
N5A—C13A—C14A—C15A	−45.4 (6)	C14D—C15D—C16B—C15B	−53.6 (18)
C14C—C13A—C14A—C15A	48.0 (9)	C14B—C15B—C16B—N5B	−39.3 (9)
C13A—C14A—C15A—C16A	54.5 (7)	C14B—C15B—C16B—C15D	67 (2)
N5A—C13A—C14C—C15C	15.2 (9)	C20B—N6B—C17B—C18B	−2.4 (9)
C14A—C13A—C14C—C15C	−76.9 (13)	P2B—N6B—C17B—C18B	158.3 (9)
C13A—C14C—C15C—C16A	−20.5 (10)	C20B—N6B—C17B—C18D	−23.0 (7)
C13A—N5A—C16A—C15C	−9.8 (6)	P2B—N6B—C17B—C18D	137.8 (6)
P2A—N5A—C16A—C15C	−164.7 (5)	N6B—C17B—C18B—C19B	−2.6 (16)
C13A—N5A—C16A—C15A	11.7 (5)	C18D—C17B—C18B—C19B	83 (3)
P2A—N5A—C16A—C15A	−143.2 (4)	C17B—C18B—C19B—C20B	6.2 (18)
C14C—C15C—C16A—N5A	17.6 (8)	N6B—C17B—C18D—C19D	47.6 (8)
C14C—C15C—C16A—C15A	−47.8 (10)	C18B—C17B—C18D—C19D	−51 (3)
C14A—C15A—C16A—N5A	−41.3 (6)	C17B—C18D—C19D—C20B	−56.8 (10)
C14A—C15A—C16A—C15C	78.6 (13)	C17B—N6B—C20B—C19B	6.7 (10)
C20A—N6A—C17A—C18C	16.7 (9)	P2B—N6B—C20B—C19B	−155.6 (9)
P2A—N6A—C17A—C18C	−180.0 (8)	C17B—N6B—C20B—C19D	−12.2 (7)
C20A—N6A—C17A—C18A	−20.0 (7)	P2B—N6B—C20B—C19D	−174.5 (6)
P2A—N6A—C17A—C18A	143.3 (6)	C18B—C19B—C20B—N6B	−7.6 (15)
N6A—C17A—C18A—C19A	44.7 (10)	C18B—C19B—C20B—C19D	51 (3)
C18C—C17A—C18A—C19A	−51.7 (17)	C18D—C19D—C20B—N6B	43.1 (9)
C17A—C18A—C19A—C20A	−53.7 (14)	C18D—C19D—C20B—C19B	−83 (3)
N6A—C17A—C18C—C19C	−29.4 (15)	P3B—N7B—C21B—O6B	−8.5 (7)
C18A—C17A—C18C—C19C	61.1 (17)	P3B—N7B—C21B—C22B	171.5 (3)
C17A—C18C—C19C—C20A	30.4 (18)	O6B—C21B—C22B—Cl8B	−1.3 (7)
C17A—N6A—C20A—C19C	1.8 (10)	N7B—C21B—C22B—Cl8B	178.7 (3)
P2A—N6A—C20A—C19C	−162.7 (9)	O6B—C21B—C22B—Cl9B	−122.8 (5)
C17A—N6A—C20A—C19A	−13.1 (9)	N7B—C21B—C22B—Cl9B	57.1 (5)
P2A—N6A—C20A—C19A	−177.7 (8)	O6B—C21B—C22B—Cl7B	118.0 (5)
C18C—C19C—C20A—N6A	−19.3 (15)	N7B—C21B—C22B—Cl7B	−62.0 (4)
C18C—C19C—C20A—C19A	47 (3)	C26B—N8B—C23B—C24B	0.4 (8)
C18A—C19A—C20A—N6A	40.5 (12)	P3B—N8B—C23B—C24B	−150.8 (6)
C18A—C19A—C20A—C19C	−77 (4)	C26B—N8B—C23B—C24D	13.8 (6)
P3A—N7A—C21A—O6A	−3.8 (6)	P3B—N8B—C23B—C24D	−137.4 (5)
P3A—N7A—C21A—C22A	176.1 (3)	N8B—C23B—C24B—C25B	−0.9 (10)
O6A—C21A—C22A—Cl9A	−2.9 (6)	C24D—C23B—C24B—C25B	−37.0 (14)
N7A—C21A—C22A—Cl9A	177.1 (3)	C23B—C24B—C25B—C26B	1.1 (12)
O6A—C21A—C22A—Cl8A	−125.5 (4)	N8B—C23B—C24D—C25D	−49.4 (8)
N7A—C21A—C22A—Cl8A	54.5 (5)	C24B—C23B—C24D—C25D	98 (2)
O6A—C21A—C22A—Cl7A	116.8 (4)	C23B—C24D—C25D—C26B	61.6 (9)
N7A—C21A—C22A—Cl7A	−63.1 (4)	C23B—N8B—C26B—C25D	24.9 (7)
C26A—N8A—C23A—C24A	−11.2 (6)	P3B—N8B—C26B—C25D	176.7 (5)
P3A—N8A—C23A—C24A	−160.2 (5)	C23B—N8B—C26B—C25B	0.4 (7)
C26A—N8A—C23A—C24C	16.6 (6)	P3B—N8B—C26B—C25B	152.2 (6)
P3A—N8A—C23A—C24C	−132.4 (5)	C24D—C25D—C26B—N8B	−49.7 (8)
N8A—C23A—C24A—C25A	−12.2 (8)	C24D—C25D—C26B—C25B	41.5 (15)
C24C—C23A—C24A—C25A	−107.9 (13)	C24B—C25B—C26B—N8B	−1.0 (11)
C23A—C24A—C25A—C26A	30.8 (9)	C24B—C25B—C26B—C25D	−96 (2)
N8A—C23A—C24C—C25C	−33.0 (7)	C30B—N9B—C27B—C28D	−0.2 (7)
C24A—C23A—C24C—C25C	59.1 (9)	P3B—N9B—C27B—C28D	170.2 (6)
C23A—C24C—C25C—C26A	37.8 (9)	C30B—N9B—C27B—C28B	−26.4 (6)
C23A—N8A—C26A—C25C	7.3 (6)	P3B—N9B—C27B—C28B	144.0 (5)
P3A—N8A—C26A—C25C	157.4 (5)	N9B—C27B—C28B—C29B	51.4 (7)
C23A—N8A—C26A—C25A	30.1 (6)	C28D—C27B—C28B—C29B	−48.9 (10)
P3A—N8A—C26A—C25A	−179.8 (5)	C27B—C28B—C29B—C30B	−58.6 (8)
C24C—C25C—C26A—N8A	−28.7 (8)	N9B—C27B—C28D—C29D	−12.7 (11)
C24C—C25C—C26A—C25A	−118.1 (16)	C28B—C27B—C28D—C29D	71.9 (13)
C24A—C25A—C26A—N8A	−37.1 (8)	C27B—C28D—C29D—C30B	19.1 (12)
C24A—C25A—C26A—C25C	58.7 (11)	C27B—N9B—C30B—C29D	12.8 (7)
C30A—N9A—C27A—C28A	−16.8 (6)	P3B—N9B—C30B—C29D	−158.6 (6)
P3A—N9A—C27A—C28A	153.0 (4)	C27B—N9B—C30B—C29B	−9.9 (7)
N9A—C27A—C28A—C29A	31.7 (6)	P3B—N9B—C30B—C29B	178.8 (5)
C27A—C28A—C29A—C30A	−34.6 (7)	C28D—C29D—C30B—N9B	−18.5 (9)
C27A—N9A—C30A—C29A	−4.4 (6)	C28D—C29D—C30B—C29B	48.9 (11)
P3A—N9A—C30A—C29A	−174.8 (4)	C28B—C29B—C30B—N9B	42.4 (8)
C28A—C29A—C30A—N9A	23.8 (7)	C28B—C29B—C30B—C29D	−76.6 (14)

Symmetry codes: (i) *x*−1, *y*, *z*.

### Hydrogen-bond geometry (Å, °)

**Table:**

*D*—H···*A*	*D*—H	H···*A*	*D*···*A*	*D*—H···*A*
N1A—H1AA···Cl11	0.86	2.48	3.247 (3)	149
N7A—H7AC···Cl12	0.86	2.43	3.234 (3)	157
N4A—H4AC···Cl13	0.86	2.50	3.248 (3)	147
N1B—H1BA···Cl21	0.86	2.44	3.214 (3)	150
N7B—H7BC···Cl22	0.86	2.52	3.293 (3)	149
N4B—H4BC···Cl23	0.86	2.52	3.295 (3)	150

## References

[bb1] Burnett, M. N. & Johnson, C. K. (1996). *ORTEPIII* Report ORNL-6895. Oak Ridge National Laboratory, Tennessee, USA.

[bb2] Farrugia, L. J. (1997). *J. Appl. Cryst.* **30**, 565.

[bb3] Farrugia, L. J. (1999). *J. Appl. Cryst.* **32**, 837–838.

[bb4] Gholivand, K., Alizadehgan, A., Arshadi, S. & Firooz, A. (2006). *J. Mol. Struct.* **791**, 193–200.

[bb5] Oxford Diffraction (2006). *CrysAlis CCD* and *CrysAlis RED* Oxford Diffraction Ltd, Abingdon, England.

[bb6] Sheldrick, G. M. (2008). *Acta Cryst.* A**64**, 112–122. 10.1107/S0108767307043930 18156677

[bb7] Znovjyak, K., Moroz, O., Ovchynnikov, V., Sliva, T., Shishkina, S. & Amirkhanov, V. (2009). *Polyhedron*, **28**, 3731–3738.

[bb8] Znovjyak, K. O., Ovchynnikov, V. A., Moroz, O. V., Shishkina, S. V. & Amirkhanov, V. M. (2010). *Acta Cryst.* E**66**, m322. 10.1107/S1600536810006422 PMC2983502 21580260

